# Resolution Mediator Chemerin15 Reprograms the Wound Microenvironment to Promote Repair and Reduce Scarring

**DOI:** 10.1016/j.cub.2014.05.006

**Published:** 2014-06-16

**Authors:** Jenna L. Cash, Mark D. Bass, Jessica Campbell, Matthew Barnes, Paul Kubes, Paul Martin

**Affiliations:** 1 School of Physiology & Pharmacology, Medical Sciences, University Walk, Bristol University, Bristol BS8 1TD, UK; 2 William Harvey Research Institute, Barts and the London School of Medicine and Dentistry, Charterhouse Square, London EC1M 6BQ, UK; 3 Calvin, Phoebe, and Joan Snyder Institute for Infection, Immunity, & Inflammation, University of Calgary, Calgary, AB T2N 4N1, Canada; 4 School of Biochemistry, Medical Sciences, University Walk, Bristol University, Bristol BS8 1TD, UK; 5 Takeda Cambridge Ltd., 418 Cambridge Science Park, Milton Road, Cambridge CB4 0PZ, UK

## Abstract

Disorders of cutaneous repair can cause disability or death given that skin functions as a protective barrier against the external environment. The inflammatory response triggered by tissue damage is thought to play both positive (e.g., pathogen-killing) and negative (e.g., scarring) roles in repair [1–3]. Inflammatory resolution mediators such as chemerin15 (C15) control the magnitude and duration of the inflammatory response; however, their role in wound repair and scarring is unknown [4–8]. Here, we show that the C15 precursor, chemerin, and its receptor, ChemR23, are both upregulated after skin damage and that the receptor is expressed by macrophages, neutrophils, and keratinocytes. Dynamic live-imaging studies of murine cutaneous wounds demonstrate that C15 delivery dampens the immediate intravascular inflammatory events, including platelet adhesion to neutrophils, an important event in driving leukocyte recruitment. C15 administration indirectly accelerates wound closure while altering fibroblast-mediated collagen deposition and alignment to reduce scarring. Macrophage recruitment is restricted to the immediate wound site rather than spilling extensively into the adjacent tissue as in control wounds, and macrophage phenotype in C15-treated wounds is skewed toward a less inflammatory phenotype with reduced iNOS, increased Arginase-1, and lower wound tumor necrosis factor α (TNF-α) expression. Modulation of inflammatory resolution pathways in acute and chronic wounds may therefore provide a novel therapeutic avenue to improve repair and reduce scarring.

## Highlights

• C15 inhibits the earliest intravascular inflammatory events after wounding
• C15 skews wound macrophage phenotype
• C15 treatment reduces wound collagen fiber alignment and thus scarring
• Resolution pathways could be targeted to improve repair and reduce scarring

## Graphical Abstract

**Figure:**
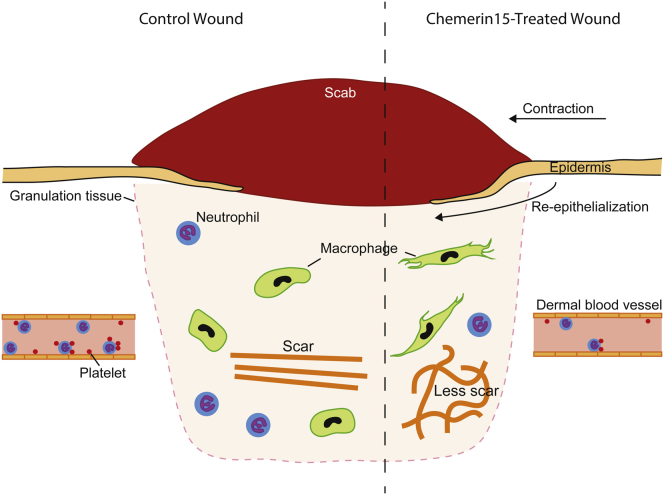

## Teaser

Cash et al. demonstrate that the resolution mediator chemerin15 reprograms the wound microenvironment through direct and indirect effects on leukocytes and stromal cells to promote repair and reduce scarring.

## Results

Repair of adult cutaneous wounds is a complex process that restores cellular structures and tissue layers but culminates in a scar consisting of linear bundles of collagen as opposed to the more randomly oriented collagen bundles found in normal skin. Wound repair in embryos involves very little inflammation and scar-free healing, suggesting that inflammatory cells are causally involved in scarring [1, 2, 9]. Resolution mediators function to dampen the inflammatory response and promote its resolution [5, 10]; however, their potential role in the context of dermal wound repair has, surprisingly, barely been investigated. Studies have shown that mice lacking the resolution mediator annexin A1 suffer delayed wound closure [11] and that proresolving mediators, including resolvin E1 (RvE1), promote corneal re-epithelialization and organ regeneration [12, 13].

The anti-inflammatory and proresolving peptide chemerin15 (C15) is a 15 aa peptide derived from cleavage of chemerin, which promotes phagocytosis of microbes and inhibits heart damage after myocardial infarction in a mouse model [6–8]. Full-length chemerin is a proinflammatory protein that can be cleaved to generate antimicrobial peptides and anti-inflammatory species [8, 14, 15]. C15 acts through the G-protein coupled receptor ChemR23, expressed on macrophages and neutrophils [6, 16]. ChemR23 is a multifunctional receptor that transduces the anti-inflammatory and proresolving effects of C-terminal chemerin peptides, including C15 and the lipid RvE1, as well as the proinflammatory effects of full-length chemerin [17–19]. The aim of this study was to determine what role C15 might play in regulating the wound inflammatory response and how this might influence subsequent skin repair.

### Chemerin and ChemR23 Expression during Wound Healing

**Figure 1:**
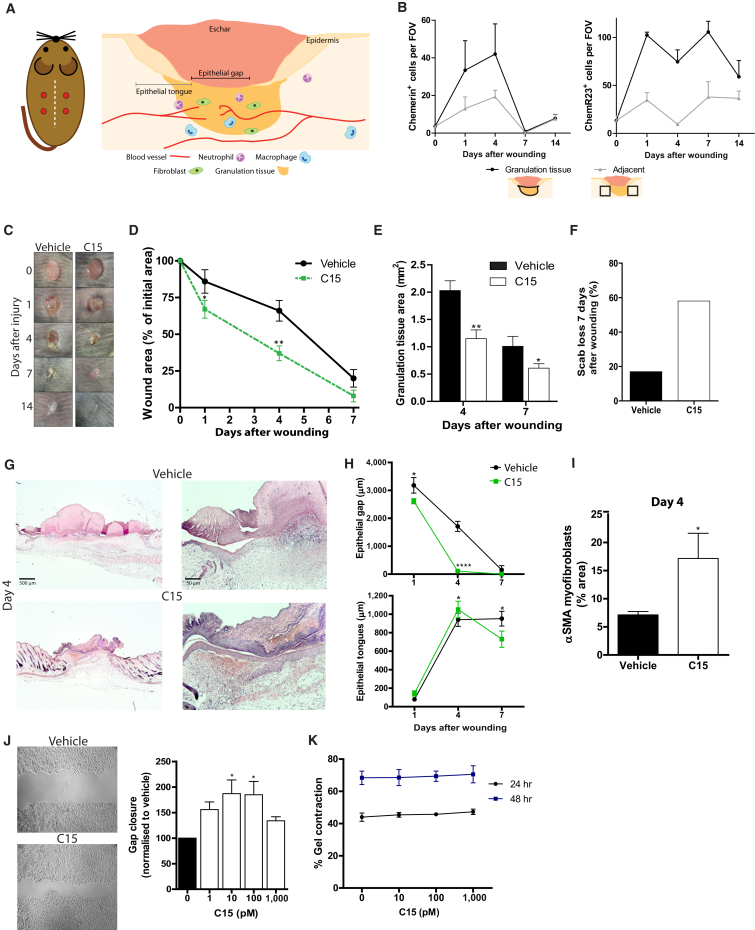
Skin Wound Healing Is Accelerated with C15 Treatment through Direct and Indirect Mechanisms Four 4 mm excisional wounds were made to the dorsal skin of Sv129Ev mice. Vehicle or C15 (100 pg/wound) in 30% Pluronic gel was administered directly into the wound immediately after wounding. (A) Schematic diagrams illustrating the location of skin wounds and measurements derived from histological sections. (B) Immunohistochemical staining during the wound repair time course revealed increases in the number of cells expressing chemerin and ChemR23 after wounding, particularly in granulation tissue. (C) Macroscopic photos of vehicle- and C15-treated wounds 0–14 days after wounding. (D) Wound area relative to initial wound area at days 1, 4, and 7 after wounding. (E) Granulation tissue area at wound midpoints. (F) Scab loss 7 days after wounding. (G) Representative photos of sections from day 4 wound midpoints stained with haematoxylin and eosin. (H) Re-epithelialization quantified by measurement of the length of wound epithelial gaps and tongues on days 1, 4, and 7 after wounding. (I) αSMA myofibroblasts in day 4 wounds. (J) HaCaTs (human keratinocytes) were allowed to migrate for 15 hr to fill in a “scratch wound” in vitro in the presence of C15 (1–1,000 pM) or media control. (K) Murine dermal fibroblasts were suspended in a collagen I and media mixture supplemented with 10–1,000 pM C15 or vehicle (media) control. Gels were allowed to solidify at 37°C, and gel contraction was assessed 24 and 48 hr later. Data are expressed as means ± SEM; there were six to ten mice (D and F) or four to eight wounds (B, E, H, and I) per treatment group or four independent experiments (J and K). ^∗^p < 0.05, ^∗∗^p < 0.01, and ^∗∗∗^p < 0.001 relative to vehicle-treated controls. See also Figure S1. The following abbreviation is used: FOV, field of view.

Our full-thickness 4 mm excisional wounding model involves the complete removal of the epidermis, dermis, and panniculus carnosus, which is firmly adherent to the base of the dermis. Healing commences after the formation of a fibrin clot that is invaded by granulation tissue and by the migration of an epidermal tongue along the interface between the granulation tissue and the clot (schematic in Figure 1A).

We found few chemerin^+^ (C15 precursor) cells in unwounded skin, but their numbers increased profoundly in granulation tissue—they peaked at days 1 and 4 after wounding before declining to negligible numbers by day 7 (Figure 1B). Macrophages, but not neutrophils, were found to be chemerin^+^ (Figure S1, available online). ChemR23 was upregulated 10-fold in granulation tissue by day 1 and remained high until day 7 (Figure 1B); it was expressed by wound macrophages, neutrophils, and keratinocytes, but not fibroblasts (Figure S1).

### C15 Improves the Rate and Quality of Wound Repair

To investigate the potential effect of the chemerin peptide C15 on wound repair, we administered peptide (100 pg/wound) or vehicle control in Pluronic gel immediately after wounding. Macroscopic analysis showed that wound closure was markedly accelerated at all stages of repair in C15-treated wounds (Figures 1C and 1D; Figure S1) with a corresponding reduction in granulation tissue (Figure 1E). Maximal differences in wound areas were observed 4 days after wounding (C15: 36% ± 5% versus vehicle; 70% ± 6% of starting wound size; Figure 1D). We also noted that C15 treatment resulted in significantly earlier scab loss—58% of wounds lost their scabs by day 7 in comparison to only 17% of vehicle-treated wounds (Figure 1F). These differences suggest that C15 promotes faster wound re-epithelialization, which may be directly by affecting keratinocyte migration and/or indirectly by modulating fibroblast behavior and leading to accelerated wound contraction. Indeed, epithelial gaps (schematic in Figure 1A) constituted only 4% of the width of day 4 C15-treated wounds in comparison to 60% of vehicle-treated wounds; however, epithelial tongues were only fractionally longer (Figures 1G and 1H), suggesting that quicker re-epithelialization is largely a consequence of a reduced denuded area available for re-epithelialization. Indeed, αSMA staining for contractile myofibroblasts (Figure 1I) indicated that day 4 C15-treated wounds possessed higher levels of these contractile cells than did control wounds. Interestingly, *ChemR23*^−/−^ mice showed elevated neutrophil recruitment, but no significant changes in wound re-epithelialization or closure (Figure S2).

### C15 Exerts Direct and Indirect Effects on Keratinocyte and Fibroblast Physiology

While our studies demonstrate that C15 can promote wound re-epithelialization and closure, it is unclear whether this is mediated entirely through C15’s effects on inflammation or whether there might be some direct modulation of keratinocyte and fibroblast behavior by the peptide. We found that C15 modestly promoted keratinocyte migration into an in vitro “scratch wound” with optimal doses of 10–100 pM. (Figure 1J). However, C15 had no effect on fibroblast-mediated collagen gel contraction (Figure 1K). These data suggest that the accelerated wound closure observed in C15-treated wounds is via indirect mechanisms, whereas accelerated re-epithelialization could, in part, be through direct effects of C15 on keratinocytes.

### C15 Regulates Neutrophil and Platelet Behavior Immediately after Wounding

Wound repair from the late stages of fetal development onward is accompanied by robust recruitment of inflammatory cells (neutrophils and monocytes) to the wound [20]. The optimal balance between sufficient leukocyte recruitment to combat any invading microorganisms and excessive pathological inflammation that contributes to scarring is clearly of critical importance in any tissue-repair response or therapeutic treatment [2, 20].

Platelets are essential for primary hemostasis, but they are also important amplifiers of acute inflammation. Activated platelets can trigger neutrophil recruitment by directly interacting with neutrophils to prompt a process called “secondary” neutrophil capture [21–23]. Platelets have previously been shown to express ChemR23 and respond to the ChemR23 lipid ligand RvE1 [24, 25], but the potential role of C15 in platelet biology has not been tested.

**Figure 2:**
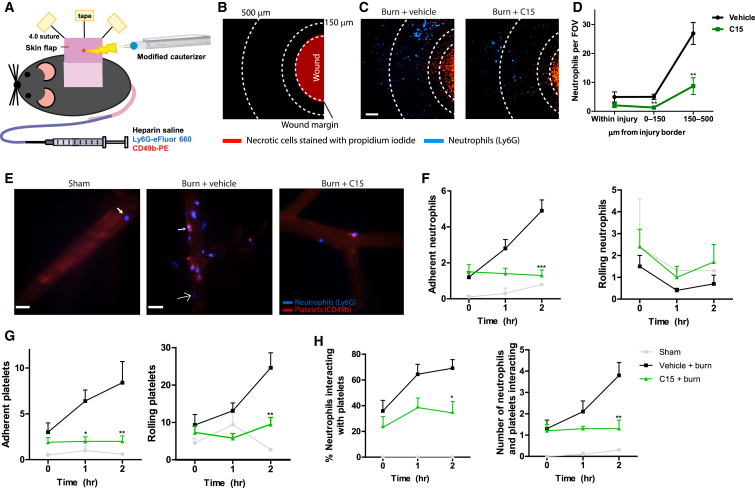
C15 Inhibits Early Platelet and Neutrophil Inflammatory Events after Cutaneous Wounding A single 580 ± 39 μm^2^ focal injury was induced on the surface of a dorsal skin flap with a modified electrocautery device. C15 (100 pg/wound) or vehicle (saline) was administered intradermally immediately after wounding. For sham experiments, mice were prepared for intravital microscopy and imaged identically to injured animals, but no injury was induced. (A) Schematic diagram of the skin preparation with burn injury for multichannel fluorescence spinning-disk confocal microscopy. (B) Schematic showing the wound as viewed with a 4× objective; necrotic cells stained with propidium iodide are shown in red. (C) Low-power (4×) views of necrotic cells (propidium iodide, red) and neutrophil recruitment (blue) to vehicle- or C15-treated wounds 2 hr after injury. The scale bar indicates 150 μm. Dotted lines indicate the wound margin, 150 μm and 500 μm from the wound edge. (D) Quantification of neutrophil recruitment to wounds treated with vehicle or C15. (E) High-power (20x) views of neutrophil (Ly6G, blue) and platelet (CD49b, red) behavior within dermal postcapillary venules 2 hr after wounding. The scale bar indicates 30 μm. The arrow in “Sham” indicates a rolling neutrophil. The large arrow in “Burn + vehicle” indicates a neutrophil without interacting platelets. The small arrow indicates neutrophils with interacting platelets. (F–H) Quantification of neutrophil rolling and adhesion (F), platelet rolling and adhesion (G), and platelet-neutrophil interactions (H) after wounding. Data are expressed as means ± SEM; four to eight vessels were visualized per time point in each mouse, and there were four to eight animals per treatment group. Vehicle (n = 4), burn (n = 8), C15 (n = 7). ^∗^p < 0.05, ^∗∗^p < 0.01, and ^∗∗∗^p < 0.001 relative to vehicle-treated mice. See also Movies S1, S2, and S3. The following abbreviation is used: FOV, field of view.

The standard excisional wound model described above is not amenable to live imaging of the dynamics of wound leukocyte and platelet behavior. To visualize the immediate inflammatory events occurring after cutaneous wounding, we used fluorescence spinning-disk intravital microscopy of small cutaneous burn wounds treated with vehicle or C15 (schematics in Figures 2A and 2B [26]).

At 2 hr after wounding, the first neutrophils were seen entering the wound, and the majority were found 150–500 μm from the edge of the burn. Even at this early stage, the effects of C15 were clear: we observed a 68% reduction in neutrophil numbers both within the wound and in the surrounding area (up to 500 μm; Figures 2C and 2D). Sham-operated animals exhibited low levels of neutrophil-endothelial cell interactions (Figures 2E and 2F), platelet-endothelial cell interactions (Figure 2G), and neutrophil-platelet interactions (Figure 2H). However, tissue injury elicited marked platelet and neutrophil rolling and adhesion within nearby postcapillary venules. Treatment with C15 significantly blunted these inflammatory responses. Specifically, 2 hr after burning, neutrophil adhesion was reduced by 74% (Figure 2F), platelet adhesion and rolling were inhibited by 76% and 62%, respectively (Figure 2G), and the percentage of neutrophils interacting with platelets dropped by 49% (Figure 2H; see also Movies S1, S2, and S3).

### C15 Restricts Leukocyte Recruitment and Modulates Macrophage Phenotype during Repair

**Figure 3:**
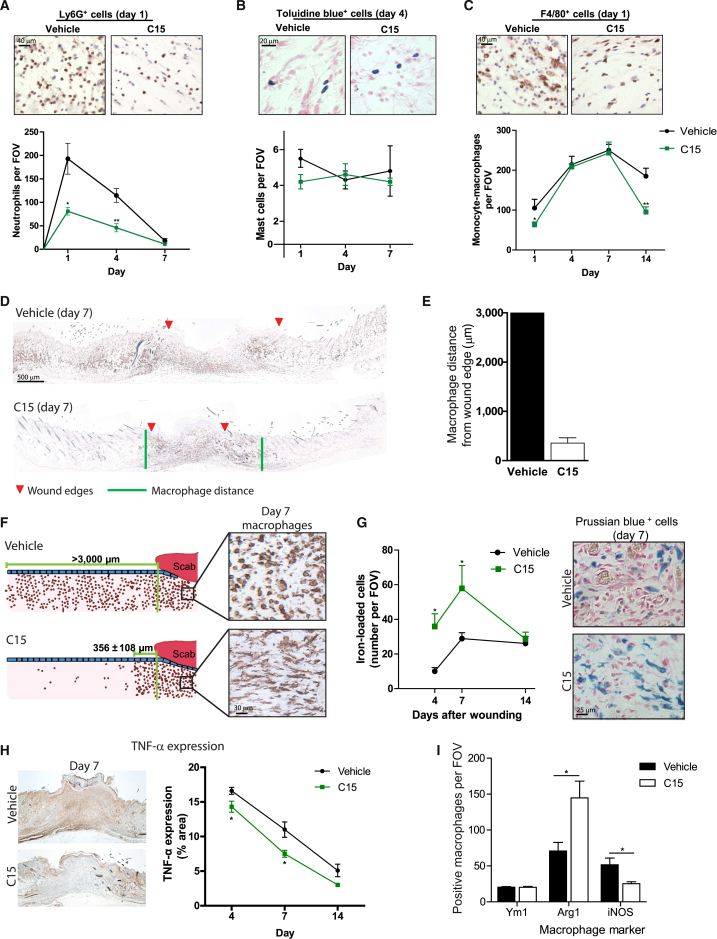
C15 Dampens Wound Leukocyte Recruitment and Skews Macrophage Phenotype Four 4 mm excisional wounds were made to the dorsal skin of Sv129Ev mice. Vehicle or C15 (100 pg/wound) in 30% Pluronic gel was administered directly into the wound immediately after wounding. (A–C) Representative micrographs and quantification of neutrophil (Ly6G^+^ cell, brown) (A), mast cell (toluidine blue^+^ cell, blue) (B), and monocyte-macrophage (F4/80^+^ cell, brown) (C) recruitment to wound granulation tissue up to 14 days after wounding. (D) Macrophage immunostaining through the midpoint of day 7 wounds. Red arrows indicate wound edges, and green lines mark the edge of the macrophage-dense area. (E) Quantification of the distance that macrophages extend beyond the wound margin into the surrounding tissue. (F) Schematic showing macrophage distribution around vehicle- and C15-treated day 7 wounds and corresponding photos of macrophage morphology. (G) Quantification and representative photos of Prussian blue^+^ iron-loaded cells within the wound. (H) Quantification and representative photos of TNF-α expression (brown). (I) Characterization of macrophage phenotype within day 7 C15- and vehicle-treated wounds. Ym1 and Arg1 are M2 markers, and iNOS is an M1 macrophage marker. Data are expressed as means ± SEM; there were four to eight wounds per treatment group. ^∗^p < 0.05 and ^∗∗^p < 0.01 relative to vehicle-treated wounds. The following abbreviation is used: FOV, field of view.

In the excisional wounding model, we monitored leukocyte recruitment dynamics during the repair period. C15 administration inhibited neutrophil recruitment to excisional cutaneous wounds by up to 60% on day 1 (50% on day 4; Figure 3A), but no changes in mast cell numbers were observed (Figure 3B). C15 reduced monocyte-macrophage recruitment on day 1 by 40%, but by day 7, there appeared to be no significant difference in the numbers of F4/80^+^ cells at the wound site (Figure 3C). However, more careful spatial analysis revealed a markedly different macrophage distribution in the immediately adjacent unwounded tissue (Figure 3D). Unlike vehicle-treated day 7 wounds, in which F4/80^+^ cells were abundant up to 3,000 μm from the wound edge, C15-treated wounds showed restricted monocyte-macrophage recruitment to the wound site such that macrophages extended only 356 ± 108 μm into the adjacent tissue (Figures 3D–3F). This suggests that C15 may function to prevent excessive macrophage recruitment, which could be detrimental to the wound repair process, and to direct macrophages to leave the wound earlier. Indeed, by day 14, markedly fewer F4/80^+^ cells were detectable at the wound site. This may be of significant potential therapeutic benefit since spreading of the inflammatory response beyond the immediate wound site may lead to excessive scarring, as seen in extreme cases with keloid scars [27].

We also noted that a high proportion of vehicle-treated wound macrophages exhibited a round morphology, whereas C15-treated wound macrophages had many protrusions, which may reflect a more migratory or phagocytic phenotype (Figure 3F). Indeed, C15-treated wounds exhibited higher levels of Prussian blue^+^ iron-loaded cells (macrophages with phagocytosed erythrocytes) at days 4 and 7 after wounding, and these cells appeared to track away from the wound (Figure 3G). Increased wound haem clearance could aid repair by removing a known stimulus of “M1” macrophage activation, which has previously been associated with poor repair in iron-burdened chronic wounds [28]. Analysis of tumor necrosis factor α (TNF-α) expression revealed markedly lower levels within the wound granulation tissue after C15 treatment, suggestive of a less inflamed environment (Figure 3H). Moreover, phenotypic analysis of wound macrophages showed that more macrophages in C15-treated wounds expressed Arginase-1, typically associated with an “M2” phenotype, and fewer expressed iNOS, typically associated with an “M1” phenotype, although no change in the “M2 marker” Ym1 was observed (Figure 3I).

Collectively, these data indicate that C15 administration not only acts to restrict inflammatory cell recruitment to the wound site but may also direct earlier resolution and skew macrophage phenotype toward that typically linked with improved wound repair.

### C15 Inhibits Scarring by Modifying Collagen Organization within the Healing Wound

Collagen is a major structural component of normal skin, and prior to wounding, it exhibits a basketweave appearance in the dermis, whereas after healing, collagen is thought to be deposited in aligned bundles characteristic of a scar. The degree of scarring has previously been assessed either by measurement of collagen levels within the wound or by subjective measurement of collagen fiber orientation [29, 30].

**Figure 4:**
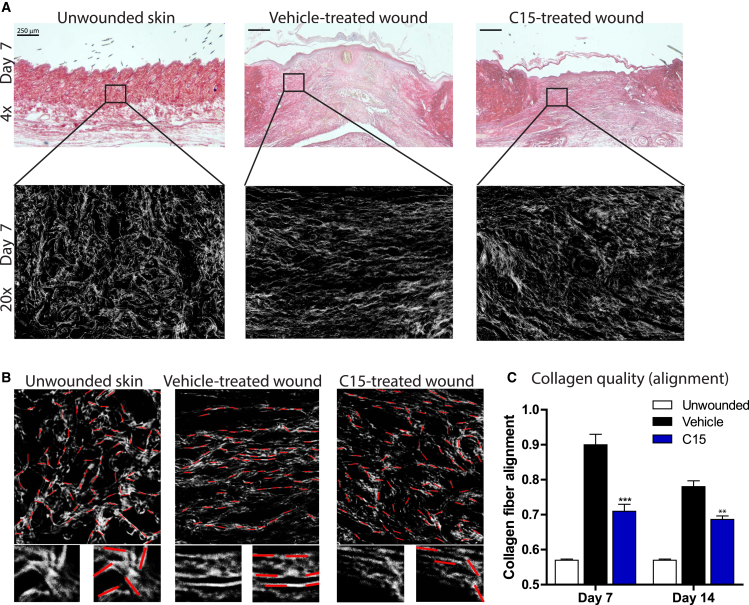
C15 Inhibits Scar Formation (A) Day 7 wound midsections (4× objective on top; 20× objective on bottom) stained with picrosirius red (collagen) from unwounded skin and vehicle- and C15-treated wounds. (B) Images used for algorithm analysis (zoomed-in areas are shown underneath). Red lines were fitted by the algorithm along white lines of collagen, and the angles of each line were measured and compared for determining fiber alignment. (C) Quantification of collagen fiber alignment in day 7 and 14 wounds. An entirely randomly oriented network of collagen fibers would score 0.5 in this analysis, whereas complete fiber alignment would score 1. Data are expressed as means ± SEM; there were six wounds per treatment group. ^∗∗∗^p < 0.001 and ^∗∗^p < 0.01 relative to vehicle-treated wounds. See also Figure S3 and Supplemental Experimental Procedures.

To better assess collagen fiber orientation, we designed a novel algorithm to quantify collagen fiber alignment within wounded versus unwounded dermis (Figure S3). As expected, unwounded skin displayed abundant collagen with a basketweave appearance (largely randomly oriented fibers) with a low alignment (0.57 ± 0.003; Figures 4A–4C). In vehicle-treated day 7 wounds, collagen was laid down in a more linear fashion, resulting in a high alignment of 0.90 ± 0.01. Wound treatment with C15, however, resulted in collagen deposition with a more random collagen fiber orientation closer to unwounded dermal collagen organization, which equated to reduced alignment in comparison to that of vehicle-treated wounded skin (C15: 0.71 ± 0.02; Figures 4A–4C). The data obtained from our alignment algorithm compare favorably with those of an established technique for assessing collagen quantity by viewing wounds stained with picrosirius red under polarized light (Figure S3) and suggest that a downstream consequence of C15 treatment appears to be a reduction in the degree of scarring at repair sites.

## Discussion

Various genetic and pharmacological approaches for knocking down the wound inflammatory response have suggested that leukocytes deliver signals that lead to scarring [2, 31]. Clearly, genetic deletion of immune cells is not a practical clinical option to control scarring; however, since resolution mediators drive the cessation of the inflammatory response, they may provide therapeutic targets for modulating wound inflammation in an attempt to regulate the repair and scarring process [10, 32].

In this study, we assessed the impact of a proresolving mediator on dermal wound repair and scarring. By administering the anti-inflammatory and proresolving peptide C15 to wounds, we were able to speed up wound closure, re-epithelialization, and scab loss while suppressing wound neutrophil and macrophage recruitment and limiting scarring. C15 most likely achieves these effects through direct modulation of leukocyte dynamics at the wound site since our previous studies have demonstrated that C15 can suppress activation of integrins required to mediate neutrophil adhesion and transendothelial migration [6]. This may, in part, be the mechanism underpinning our observed reduction in neutrophil recruitment to excisional wounds (Figure 3A) and dampened neutrophil-endothelial and neutrophil-platelet interactions at burn lesions (Figure 2). Similar observations have been made by others with the resolution mediator resolvin D2 [33]. Furthermore, C15 stimulates a switch of macrophage phenotype to cells expressing M2-like markers, including arginase 1, and reduced expression of M1 markers, including iNOS and TNF-α. The resolution mediators RvE1 and maresin have also recently been shown to be capable of switching macrophage phenotype, suggesting that a defining characteristic of proresolving mediators may be their ability to switch macrophage phenotype [34].

C15 does not promote wound closure by directly regulating fibroblast physiology but rather does so through indirect actions. We postulate that leukocytes exposed to C15 in the wound bed signal to fibroblasts either through a soluble mediator or via cell-cell contacts to promote conversion to contractile myofibroblasts and thus faster contraction of the wound.

In addition, we suggest a role for C15 in regulating platelet behavior at the site of tissue damage. Platelets play a plethora of roles in physiology, but a definitive role in wound repair has yet to be established. It is known that platelets drive wound neutrophil and macrophage recruitment and secrete transforming growth factor β (TGF-β), and thus it would be tempting to assume that since wound leukocyte recruitment and TGF-β drive scarring, platelets, by proxy, might contribute to scarring. Our live-imaging studies indicate that one step where this might be driven is by platelet “capturing” of neutrophils in vessels near the wound and that C15 treatment inhibits this process.

Although it has been known for some time that collagen in scars exhibits a linear, bundled arrangement while unwounded skin exhibits a looser, basketweave appearance, very little is known about the dynamics of collagen deposition in wounds and how the behavior of fibroblasts is altered to lead to these very different patterns of collagen deposition. What is clear is that signals from inflammatory cells at the wound site directly influence these fibroblast behaviors, and thus by modulating the wound inflammatory response, it is possible to dramatically modulate the scarring phenotype. This, to a large degree, is what we have achieved through application of C15. A next step will clearly be to investigate further how immune cells change fibroblast behaviors because this will provide further therapeutic targets for blocking scarring downstream of immune regulators.

Although the cellular and molecular mechanisms behind repair and scar formation have yet to be fully elucidated, this study suggests that targeting the body’s endogenous resolution mechanisms could provide a potential therapeutic route to promote repair and minimize scarring through modulation of leukocyte and stromal cell physiology.

## Experimental Procedures

All experiments were conducted with approval from the local ethical review committee at the University of Bristol and in accordance with the UK Home Office regulations (Guidance on the Operation of Animals, Scientific Procedures Act, 1986). Wild-type (Sv129Ev) and *ChemR23*^−/−^ mice were supplied by Takeda Pharmaceuticals and used for excisional wounding studies. C57Bl6/J mice were purchased from Jackson Laboratories (Canada) and used for intravital imaging experiments.

### C15

C15 (AGEDPHGYFLPGQFA) was synthesized by Mimotopes, reconstituted (1 mM in PBS and 0.1% BSA), and stored at −80°C for up to 6 months. Peptide was diluted from fresh aliquots for each experiment.

### Excisional Cutaneous Wounding

Mice (7–10 weeks old) were randomly assigned a treatment group and anaesthetized with isofluorane. Four full-thickness excisional wounds (4 mm biopsy punch, Kai Industries) were made to the shaved dorsal skin. Vehicle (PBS) or C15 (100 pg/wound) was delivered topically by pipette into the wound cavity immediately after wounding (40 μl in a vehicle of 30% Pluronic F-127 gel [is liquid at 4°C but solidifies at body temperature]; Sigma Aldrich). Wounds were photographed with an Olympus camera and calibration card on days 0, 1, 4, 7, and 14 after wounding, and wound areas were calculated with ImageJ software.

### Preparation and Histological Analysis of Excisional Cutaneous Wounds

Wounds were harvested, prepared, and analyzed essentially as previously described [35] (for detailed methods, see the Supplemental Experimental Procedures). Because of the lack of a commercially available anti-ChemR23 antibody suitable for formalin-fixed, paraffin-embedded immunohistochemistry, we immunostained for β-galactosidase (2 μg/ml, Invitrogen) in *Cmklr1*^−/−^ LacZ reporter mice to determine the ChemR23 expression profile.

### Intravital Imaging of Cutaneous Focal Necrotic Injury

Skin preparation for intravital microscopy was performed essentially as previously described [26] (for further detail, see the Supplemental Experimental Procedures)—C15 or vehicle control was administered intradermally immediately after wounding.

### Collagen Alignment

We designed an algorithm to quantify the level of collagen fiber alignment within tissue sections stained with picrosirius red. We used 2D fast Fourier transformations to scan images for nonrandom, noncircular regions and to measure the angle of fitted ellipses. The algorithm quantified the alignment of fibers by comparing each angle with every other from an image such that we gave parallel fibers a score of 1 and perpendicular fibers a score of 0. Two perpendicular lines have an alignment of 0; therefore, in a random arrangement, total alignment = 0.5, and in perfectly aligned tissue, alignment = 1. We performed the analysis by using our novel algorithm in ImageJ software and Silverlight software. For further details, see the Supplemental Experimental Procedures and Figure S3.

### Statistical Analysis

Student’s t test and one- and two-way ANOVA with the Bonferroni post hoc test were performed with GraphPad Prism 5.0 software.

## Author Contributions

J.L.C. conceived and planned the project, designed and performed the experiments, analyzed data, and wrote the manuscript. M.D.B. wrote software and analyzed data. J.C. performed experiments. M.B. provided wild-type and *ChemR23*^−/−^ mice. P.K. contributed to the writing of the manuscript. P.M. planned the project and wrote the manuscript.

## Supplemental Information

Document S1. Figures S1–S3 and Supplemental Experimental Procedures

Movie S1. Sham

Movie S2. Burn + Vehicle

Movie S3. Burn + C15 High-power (20×) views of neutrophil (Ly6G, blue) and platelet (CD49b, red) behavior within dermal postcapillary venules 2 hr after wounding. A single 580 ± 39 μm^2^ focal injury was induced on the surface of a dorsal skin flap with a modified electrocautery device. C15 (100 pg/wound) or vehicle (saline) was administered intradermally immediately after wounding. For sham (vehicle control) experiments, mice were prepared for intravital microscopy and imaged identically to injured animals, but no injury was induced. See also Figure 2.

Document S2. Supplemental Algorithms S1 and S2

Document S3. Article plus Supplemental Information
