# Resolution Mediator Chemerin15 Reprograms the Wound Microenvironment to Promote Repair and Reduce Scarring

**DOI:** 10.1016/j.cub.2014.06.010

**Published:** 2014-06-16

**Authors:** Jenna L. Cash, Mark D. Bass, Jessica Campbell, Matthew Barnes, Paul Kubes, Paul Martin

(Current Biology *24*, 1406–1414; June 16, 2014)

Due to an oversight during the proofing stage, the version of this article originally published online contained an erroneous Figure 1 title, “Skin Wound Healing Is Accelerated with **Chemerin Treatment** through Direct and Indirect Mechanisms.” The title should have read “Skin Wound Healing Is Accelerated with **C15 Treatment** through Direct and Indirect Mechanisms.” This error has now been corrected in both the print and online versions of the article. The journal apologizes for any confusion this error may have caused.

