# Supplementary material for: Resolution Mediator Chemerin15 Reprograms the Wound Microenvironment to Promote Repair and Reduce Scarring
Source: Curr Biol. 2014 Jun 16;24(12):1406–14. doi: 10.1016/j.cub.2014.05.006 (PMC4064685; doi:10.1016/j.cub.2014.05.006)
Supplement: Document S1. Figures S1–S3 and Supplemental Experimental Procedures [file mmc1.pdf]

Current Biology, Volume *24*

Supplemental Information

Resolution Mediator Chemerin15 Reprograms the Wound Microenvironment to Promote Repair and Reduce Scarring

Jenna L. Cash, Mark D. Bass, Jessica Campbell, Matthew Barnes, Paul Kubes, and Paul Martin

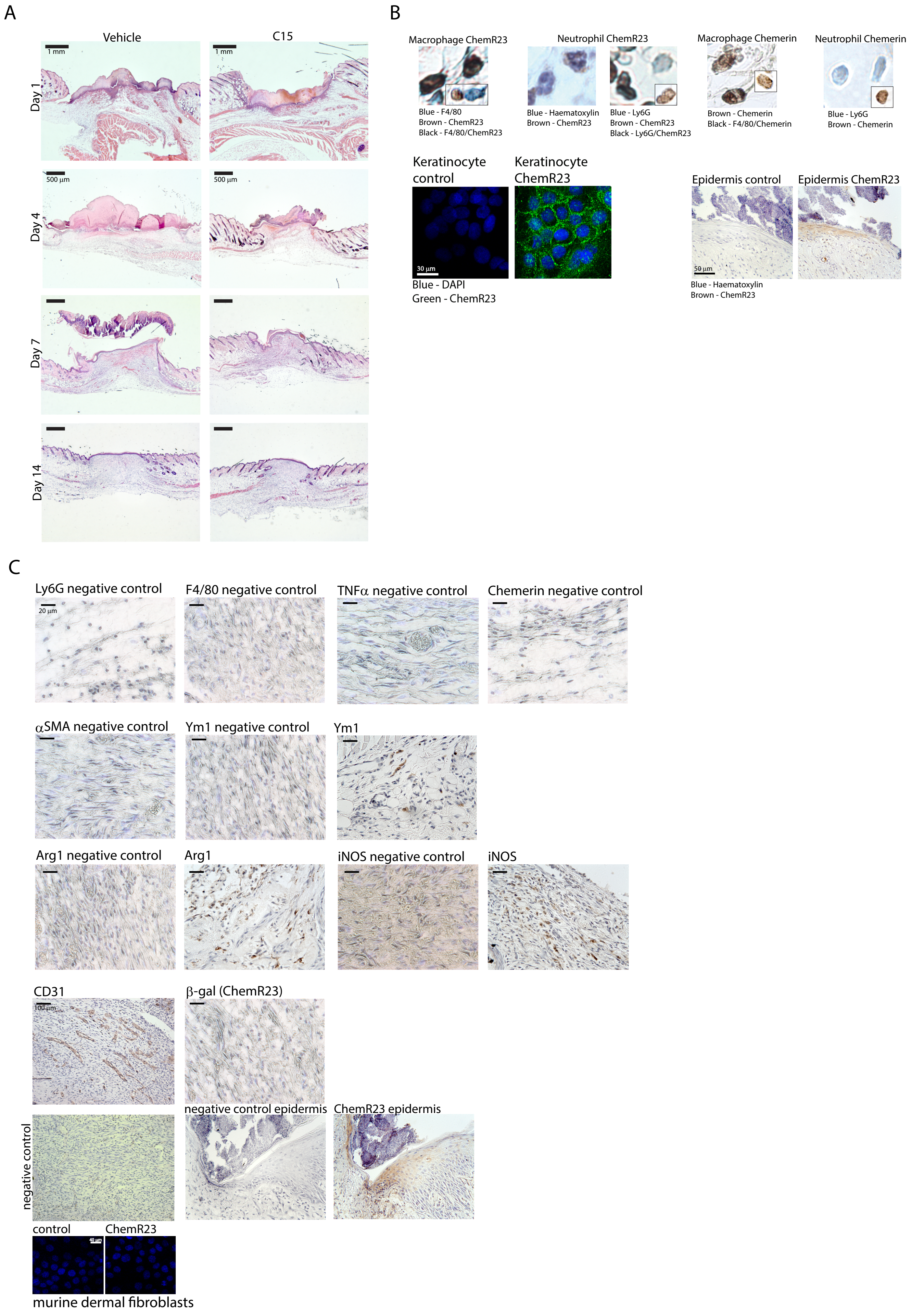

Figure S1. A Low magnification views of H&E stained wounds throughout the repair time course.
B Immunohistochemical and immunofluoresence staining to identify cell lineages expressing chemerin and ChemR23. Macrophages (F4/80+, blue), neutrophils (Ly6G and haematoxylin, blue), double staining (black/dark brown), chemerin and ChemR23 (brown). Boxes in the right hand corner of the images highlight examples of single stained cells.
C Negative controls for immunohistochemical and immunofluoresence stains. DAPI, blue. ChemR23 (Alexa 488, green or DAB, brown). See Fig.1

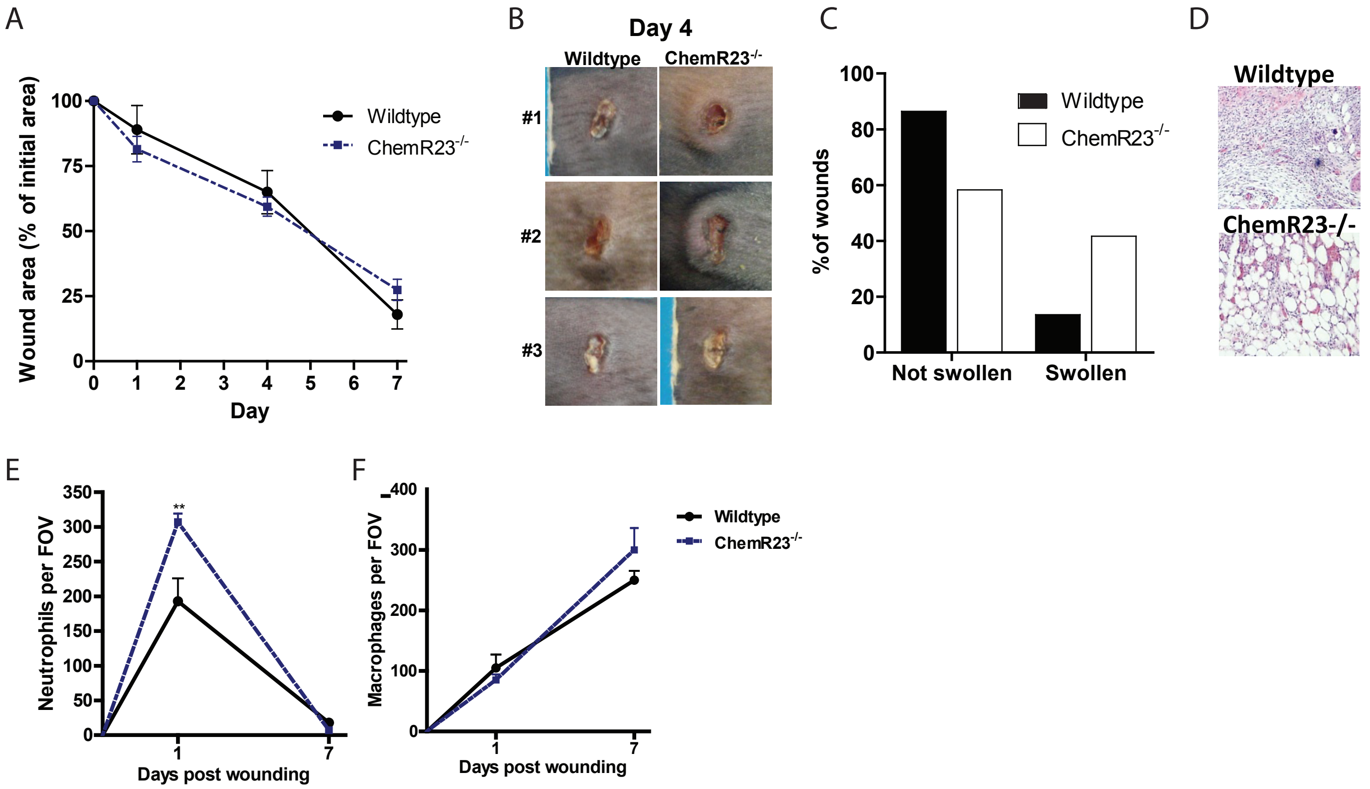

Figure S2. Wound repair in ChemR23-/- mice

A Wound area relative to initial wound area at days 1, 4 and 7 post-wounding. **B** Macroscopic photos of wildtype and ChemR23-/- wounds at day 4 post-wounding. **C** Percentage of swollen wounds in wildtype and ChemR23-/- mice 4 days post-wounding. **D** Representative haematoxylin and eosin stained wound mid-sections from day 4 wounds. **E** Neutrophil (Ly6G+ cell) and **F** macrophage (F4/80+ cell) recruitment to wound granulation tissue up to 7 days post-wounding. Data are expressed as means +/- SEM with 6 mice per experimental group (**A,C**) and 4-6 wounds per group analysed for neutrophil and macrophage recruitment. (**E,F**). **, $p<0.01$ relative to wildtype wounds. FOV, field of view. See Fig.3.

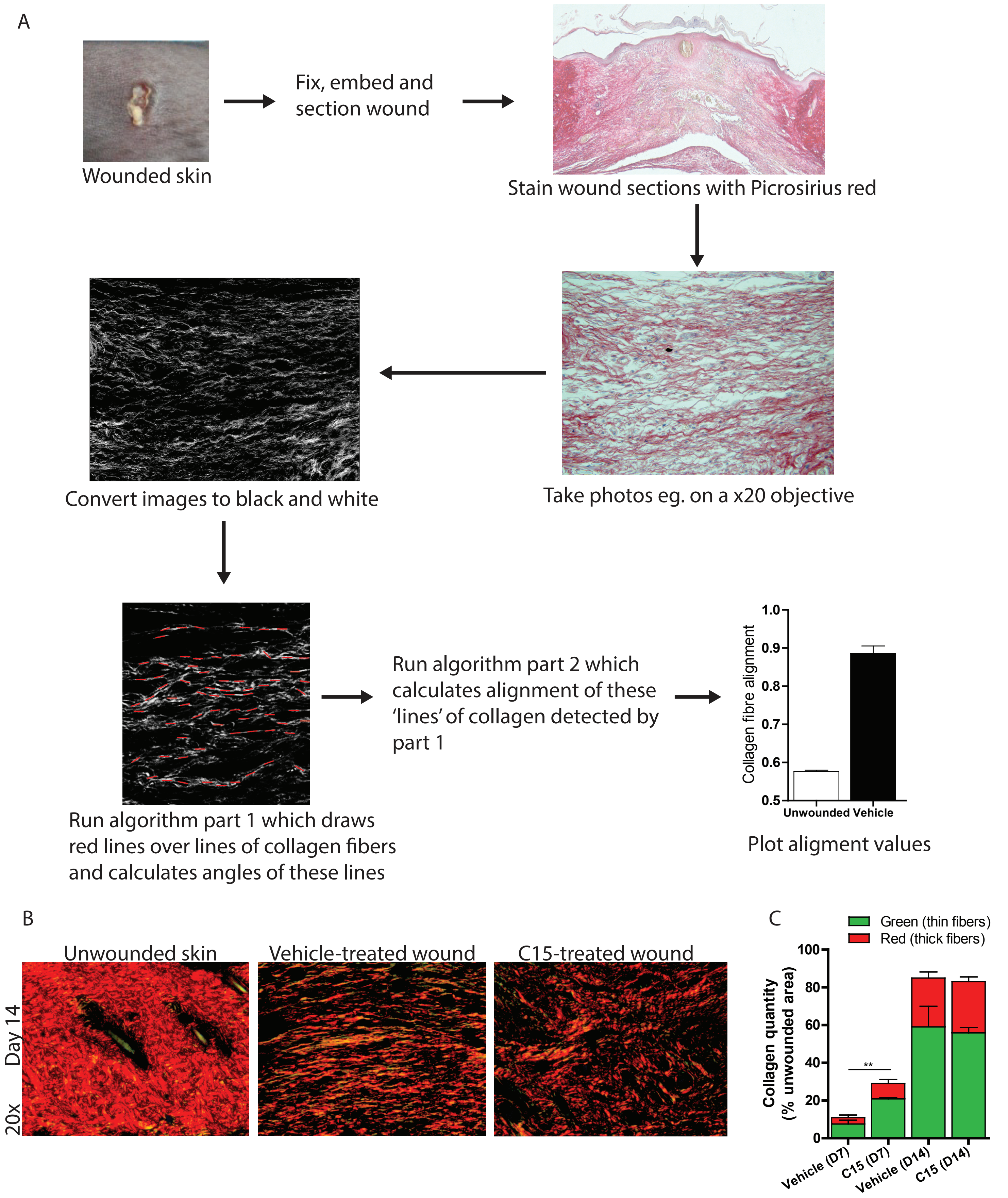

Figure S3 A Analysis of collagen alignment simplified as a flow diagram Two perpendicular lines have an alignment of 0, therefore in a random arrangement, total alingment = 0.5, and in perfectly aligned tissue, alignment = 1. **B Assessment of collagen quantity in granulation tissue during repair** Day 14 wound midsections from vehicle and C15-treated mice were stained with picrosirius red and viewed under polarised light. **C** Thick (red) and thin (green) fibers were quantified using ImageJ in day 7 and 14 wounds.
**, p<0.01 relative to vehicle treated wounds. See Fig. 4.

Supplemental Experimental Procedures

Preparation of Chemerin15 peptide

Scrambled C15 peptide (C15-S) was used in preliminary experiments to test whether PBS in pluronic gel would equally be an appropriate control. No change in wound contraction or neutrophil recruitment were observed with C15-S administration in this study. We used doses of 10 pg, 100 pg, 1 ng and 10 ng C15/wound in preliminary studies, finding that the 100 pg/wound dose achieved maximum acceleration in wound contraction and reduction in neutrophil recruitment, with no additional benefit observed with the higher doses of peptide. Peptide reconstitution was always performed under sterile conditions and reconstituted peptide is stable for approx. 6 months in -20C or -80C. Once thawed the peptide is stable on ice for several hours and is not photosensitive. When a peptide stock is thawed for use in wound repair assays it is diluted in sterile PBS and added to pluronic gel. The only step that becomes asterile is administration of the peptide to the wound. Sterile tips were used and the backs of the mice shaved and sprayed with ethanol, but wounding was not performed in aseptic conditions.

Preparation and immunohistological analysis of excisional cutaneous wounds

Wounds were harvested on days 1, 4, 7 and 14 post-wounding, fixed in 4% PFA (4 h at 4°C, Sigma) for embedding in paraffin. Day 1 and 4 wounds were cut and sections (10 μm) taken from the middle of the wound. Day 7 and 14 wounds were sectioned through the wound beyond the midpoint and wound centres identified by staining with Haematoxylin (Gills No.3) and Eosin (Sigma) and quantifying scab length (if present) and gap between severed edges of the panniculus carnosus using ImageJ software.

Proteinase K (5 μg/ml, 7 min; Dako) was used for antigen retrieval, except in the case of chemerin staining where heat-mediated antigen retrieval was performed (citrate buffer, Abcam; 95°C, 20 min). Avidin/Biotin kit, ABC kit, ABC-AP kit, DAB kit, Alkaline phosphatase Vector Blue kit, levamisole solution, biotinylated anti-rat, anti-goat and anti-rabbit secondary antibodies (all from Vector Laboratories; Peterborough, UK) were used according to the manufacturer's instructions. Numbers of leukocytes per field of view (FOV) were calculated using thresholded 32-bit images and the “analyze particles” function in ImageJ software. Numbers of chemerin+ and ChemR23+ cells were quantified similarly.

Sections were deparaffinised, rehydrated and stained with Hematoxylin (10 s), Eosin (30 s), Toluidine Blue (mast cells; 20 min, Sigma Aldrich) or Picrosirius Red (collagen; 1 h), rat anti-

mouse F4/80 (monocyte-macrophages, BM8, 2.5 μg/ml; Ebiosciences), rat anti-mouse Ly6G (neutrophils; 1A8, 2.5 μg/ml, Biolegend), rat anti-mouse polyclonal CD31 (blood vessels, 5 μg/ml; BD Biosciences), rabbit anti-mouse TNFα (2.5 μg/ml, Abcam), goat anti-mouse Ym1 (0.8 μg/ml, R&D Systems), rabbit anti-mouse arginase 1 (4 μg/ml, Santa cruz), rabbit anti-mouse iNOS (1 μg/ml, Abcam), rabbit anti-mouse β-galactosidase (2 μg/ml, Invitrogen), rabbit anti-mouse α-SMA (smooth muscle actin, 0.06 μg/ml, clone E184, Abcam) and goat-anti mouse polyclonal chemerin antibody (2 μg/ml; R&D Systems) all overnight at 4^oC.

Intravital imaging of cutaneous focal necrotic injury

Mice were anesthetized with a mixture of ketamine hydrochloride (200 mg/kg, Rogar/SBT) and xylazine hydrochloride (10 mg/kg, MTC Pharmaceuticals). After anaesthesia, the tail vein was cannulated for administration of additional anaesthetic and for injection of antibodies. Following a dorsal midline incision, the skin was dissected away from the underlying tissue yielding a skin flap that remained connected laterally with intact blood supply. The skin flap was extended over a viewing pedestal and immobilized with 4.0 silk sutures at three points around the edge of the flap. Loose connective tissue overlying the dermal microvasculature was carefully removed under a dissecting microscope.

After preparation for spinning disk intravital microscopy (SD-IVM) and immediately prior to imaging, a single 580±39 μm^2 focal injury was induced on the surface of the skin flap using the tip of a heated 30-gauge needle mounted on an electro-cautery device. C15 (100 pg) or vehicle were administered i.d immediately after wounding. For sham experiments, mice were prepared for SD-IVM and imaged identically to injured animals but no injury was induced. Following induction of necrotic injury the exposed dermal surface of the skin was covered with coverglass and continuously superfused with bicarbonate-buffered saline for the duration of the experiment. Mice were placed on a heating pad to maintain body temperature throughout experiments. Exposed tissues were visualized with an Olympus BX51 upright microscope equipped with a confocal light path (Wave-Fx; Quorum) based on a modified Yokogawa CSU-10 head (Yokogawa Electric Corporation) using a x4/0.16 UplanSApo objective or a x20/0.95W XLUMPPlanFI water immersion objective. Three laser excitation wavelengths (561-, 635- and 733 nm;Cobalt) were used in rapid succession and visualized with the appropriate long-pass filters (Semrock). Typical exposure times for excitation wavelengths were ~400 ms (561-nm), ~600 ms (635-nm) and ~900 ms (733-nm). A back-thinned EMCCD 512x512 pixel camera was used for fluorescence detection. Volocity software (Improvision) was used to drive the confocal microscope.

Neutrophils and platelets were visualized by intravenous administration of 2 μg eFLuor 660 conjugated Ly6G antibody (Ebioscience; 50-5931-82) or PE-CD49b antibody (BD Pharmingen; 558759) respectively. Blood vessels were visualized using 2 μg PerCP eFluor 710 conjugated CD31 (Ebioscience; 46-0311-82). Necrotic cells were labelled with a single application of 20 µL of 2 µM propidium iodide (Sigma Aldrich) solution to the wound.

For experiments investigating the quantity of neutrophils at sites of focal necrosis, images were acquired 2 h after injury using a 4x objective. The total number of neutrophils was then determined within specific regions of the microscopic field (within injury, 150 µm around injury, and 150-500 µm from injury border). To assess intravascular neutrophil-endothelial, platelet-endothelial and platelet-neutrophil interactions, 4-8 post-capillary venules (diameter 20-40 μm) were observed per skin prep per time point using a 20x objective and recorded for offline analysis. The extent of the inflammatory response was assessed by quantifying neutrophil rolling and adhesion, platelet rolling and adhesion and the number of neutrophils interacting with platelets in each treatment condition and time point.

Keratinocyte migration and proliferation assay

HaCaT cells (0.8 x10^6/ml) were plated into Ibidi inserts on nitric acid washed coverslips in a 24 well plate in 70 μl media (DMEM + 10% FBS, 1% Penicillin/Streptomycin). Cells were incubated for 4 h at 37°C, 5% CO_2, washed, the Ibidi insert removed and treatments (1 – 1000 pM C15) added in media. Cells were fixed 15 h later and gap closure assessed in ImageJ. Treatments were performed in sextuplicates.

Fibroblast-mediated collagen contraction assay

Murine dermal fibroblasts (a kind gift of Dr J Morris) were cultured in DMEM + 10% FBS (Gibco and Sigma) and harvested with 0.05% trypsin when 80% confluent. Cells (6 x10^4/well of a 4 well plate) were mixed with rat tail collagen I (Millipore), 10xRPMI (Gibco) and 238 mM $NaHCO_3$ (Sigma) to achieve final concentrations of 1.8 mg/ml, 1x and 24 mM respectively. Samples (600 μl) of the cell mixture were added to the wells of a 4-well tissue culture plate and spiked with 60 μl media control or C15 to achieve final concentrations of 10 pM, 100 pM and 1 nM. The collagen was allowed to polymerize at 37°C, after which the gels were gently detached from the plastic surface to allow contraction and 0.6 ml DMEM + 10% FBS containing 10 pM, 100 pM or 1 nM C15 added per well. The gels were incubated at 37°C, 5% CO_2 for 24 h and 48 h then photographs were taken.

Immunofluoresence of cultured cells

Primary dermal fibroblasts were cultured on acid washed glass coverslips coated with poly-l-lysine for 5 min. HaCaTs were cultured on glass coverslips coated with poly-l-lysine (5 min, Sigma) and coating matrix (30 min, Gibco). Cells were fixed with 4% PFA, washed with PBS, blocked with 10% goat serum for 30 min and incubated with rabbit anti-human ChemR23 (10 μg/ml, R&D Systems) or rat anti-mouse ChemR23 (20 μg/ml, Ebioscience) in 10% goat serum for 1 h at room temperature. Coverslips were washed with PBS and secondary antibody applied (goat anti-mouse Alexa 488 or donkey anti-rat Alexa 488) in 10% goat serum for 30 min. After washing coverslips with dH_2O, they were mounted in Prolong Gold Moviol containing DAPI and imaged with a confocal microscope. Controls were secondary only and isotype control with no appreciable staining detected.

Algorithm details for analysis of collagen alignment

Alignment is measured using the dot product. Each straight segment of the track defines a vector v_i, which may be compared to all the other segments of the track. The value of the dot product d_{ij} between vectors v_i and v_j is

$$d_{ij} = \frac{v_i \bullet v_j}{|v_i||v_j|}$$

If the vectors are parallel $d_{ij} = 1$, perpendicular gives $d_{ij} = 0$ and reversed gives $d_{ij} = -1$. The value of d_{ij} is then averaged over all segments, and the operation is in turn repeated for all N segments in the track to give

$$d = \frac{\sum_{i=1}^{i=N} \frac{\sum_{j=1}^{j=N} d_{ij}}{N}}{N}$$

Detailed instructions for use of the plugin to quantify collagen alignment.

(related to Figure 4 and Figure S3).

Please do not hesitate to contact jenna.cash@bristol.ac.uk and mark.bass@bristol.ac.uk for help if required.

Image processing

1) Stain wound sections with picrosirius red
2) Take eg. 20x photos of picrosirius red staining in wound granulation tissue (photos taken under polarised light should also work)
3) Save the Collalign plugin to the plugins folder in ImageJ (Disk C: -> Program Files -> Image J -> plugins)

4) Open the photo in ImageJ

5) Click Image -> Color -> Split channels. You will now have 3 images (red, green and blue)

6) There are 2 ways to generate the greyscale image for analysis. For most images, including sections only stained with picrosirius red use approach “a”. Images with a counterstain may give better definition by calculating the contrast between picrosirius red and counterstain, use approach “b”.

6a) i) Close red and blue images.

II) Select green image, click Edit -> Invert

iii) You will now have a black and white image of the collagen that looks something like the below.

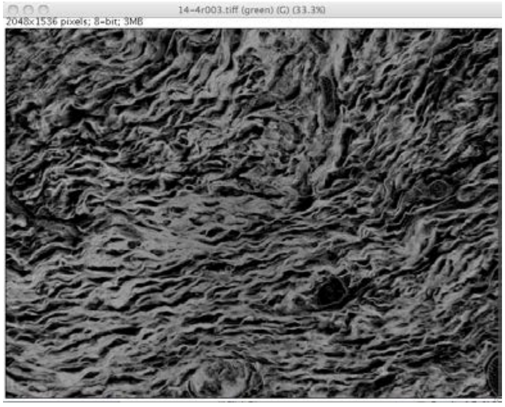

6b) i) Close green image.

ii) Click Process -> Image calculator -> subtract the blue image from the red image

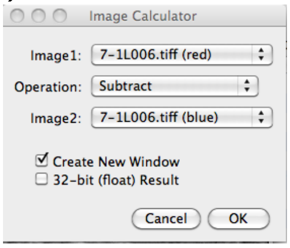

iii) You will now have a black and white image of the collagen that looks something like the below.

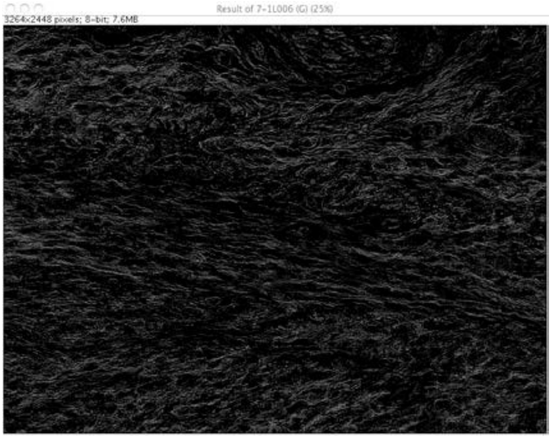

7) Click Plugins -> collagen alignment

8) The values for the presets suggested by the plugin will work for many images but can be altered if the plugin is not fitting lines well. The plugin works by scaling the

brightness of the image between minimum and maximum so should work with a range of image intensities. Adjustable variables include square size, overlapping area, mean grey value, minimal and maximum standard deviation of the grey levels. Each variable is defined in more detail at the end of the instructions.

9) The plugin will take 5-20 minutes to run, depending on the size of the image, the size of the boxes to be analysed, and whether you run overlapping boxes. You will see the analysis box moving over the image named ".tif". Each time the plugin successfully finds an intensity distribution can be added, the "orientation.tif" will come to the front and a red line will be added, and an angle value (in radians) will be added to the "log" file.

10) The "orientation.tif" image will automatically save to the location specified when starting the plugin. A zoom in of the orientation image will look like:

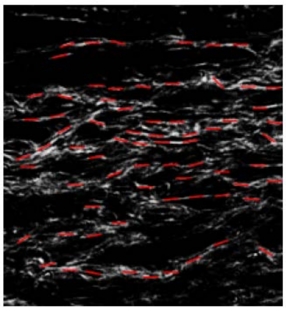

The red lines are centred in each square analysed and represent the angle of high intensity contrast across the whole square.

11) The log file will save to the same location, adding a new data set for each slice. The log file lists the angle (in radians) of each successfully fitted line. The results file is only required to run the plugin and not needed.

Alignment calculation

1) The alignment of fibres can be calculated by comparing the angle between individual lines, such that a pair of parallel lines will return an alignment value of 1 and a pair of perpendicular lines will return an alignment value of 0. This means that for perfectly aligned fibres, the total alignment value for parallel fibres will be 1 and for random fibres it will be 0.5.

2) Alignment can be calculated using the Bassalign.f90 fortran script.

3) Download and install the Silverlight compiler software: http://www.microsoft.com/silverlight/

4) From the computer programs list open:
Silverfrost -> Silverfrost FTN95 -> Plato IDE

5) In the Plato program, File -> Open -> Bassalign.f90

6) Build -> Compile

7) To create an input data file use notepad to a file in the format:

9	
1	0.0329
2	0.0336
3	-0.1201
4	0.3294
5	0.1237
6	-0.0418
7	0.1067
8	0.3015

Where 9 is the number of data points.
Where 1-9 are the sequentially numbered angle values to come out of the Collalign log file

9	0.5163	The numbers pasted across from the log file need to be followed by a couple of empty lines (hit return/enter twice).

8) Save the file as data.txt in the same place as the Bassalign.f90 file.
9) In the Plato program, Build -> Start run
10) The alignment value will be returned in a new window, along with the number of data values.

Adjustable variables are:

Square size: The plugin divides the image into squares and analyses each square in turn. Square size dictates size of the squares and can be set to 16, 32, 64, 128 or 256 pixels. Low values will find it easier to fit lines to fibres but are susceptible to noise in the image. Large values should in theory detect the strong fibres only, but often struggles to find a dominant line of intensity due to the high level of complexity in a single square. (Default = 64)

Overlapping area: The plugin divides the image into squares and analyses each square in turn. For overlap = 1, it will analyse divide up the image like a chessboard and analyse each square. For overlap = 2, the squares analysed will overlap so you will get more values from a single image, but there may be some redundancy as each pixel is being analysed twice.

***Mean grey value:** This instructs the plugin as to what value of mean grey value to expect in a square and stops the plugin fitting lines to noise values in very dim squares.

***Minimal standard deviation of the grey levels:** This ensures that the plugin is fitting lines to stained fibres, rather than noise in a square. Reducing the value will enable the plugin to find more squares, but generate more erroneous fits.

***Maximal standard deviation of the grey levels:** This stops the plugin from analysing artificial structures, such as air bubbles in the image. The brightness of genuine collagen fibres should fall within limited boundaries.

Parameters marked with an asterisk can be altered to improve sensitivity and fit more lines, or reduce erroneous fits.

Note: Up to 9 images can be converted into a single stack, and the plugin will analyse each slice in turn, saving the outputs as it goes.
