# Supplementary figures and images for: Resolution Mediator Chemerin15 Reprograms the Wound Microenvironment to Promote Repair and Reduce Scarring
Source: Curr Biol. 2014 Jun 16;24(12):1406–14. doi: 10.1016/j.cub.2014.05.006 (PMC4064685; doi:10.1016/j.cub.2014.05.006)

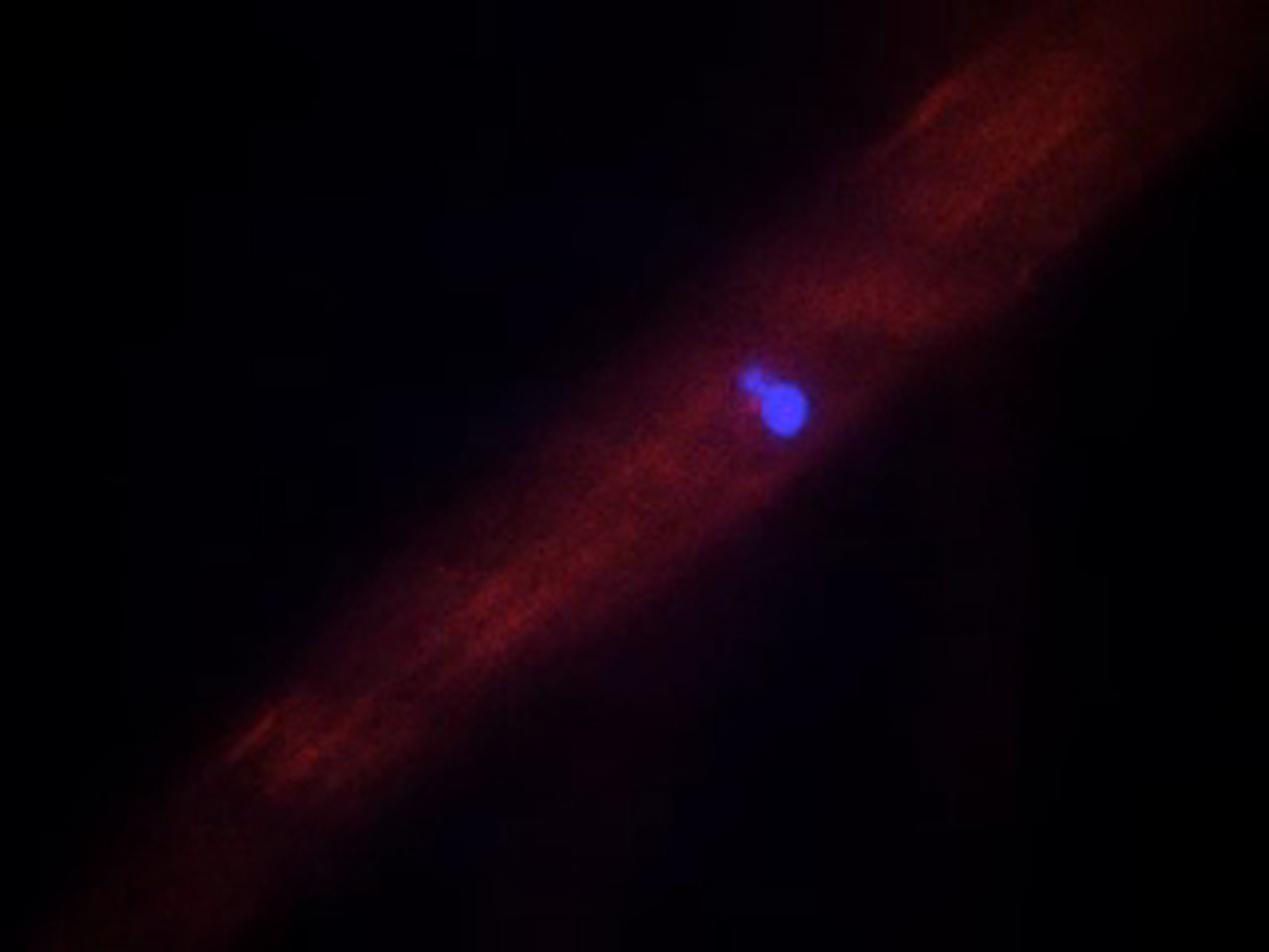

Supplement: Movie S1. Sham [file mmc2.jpg]

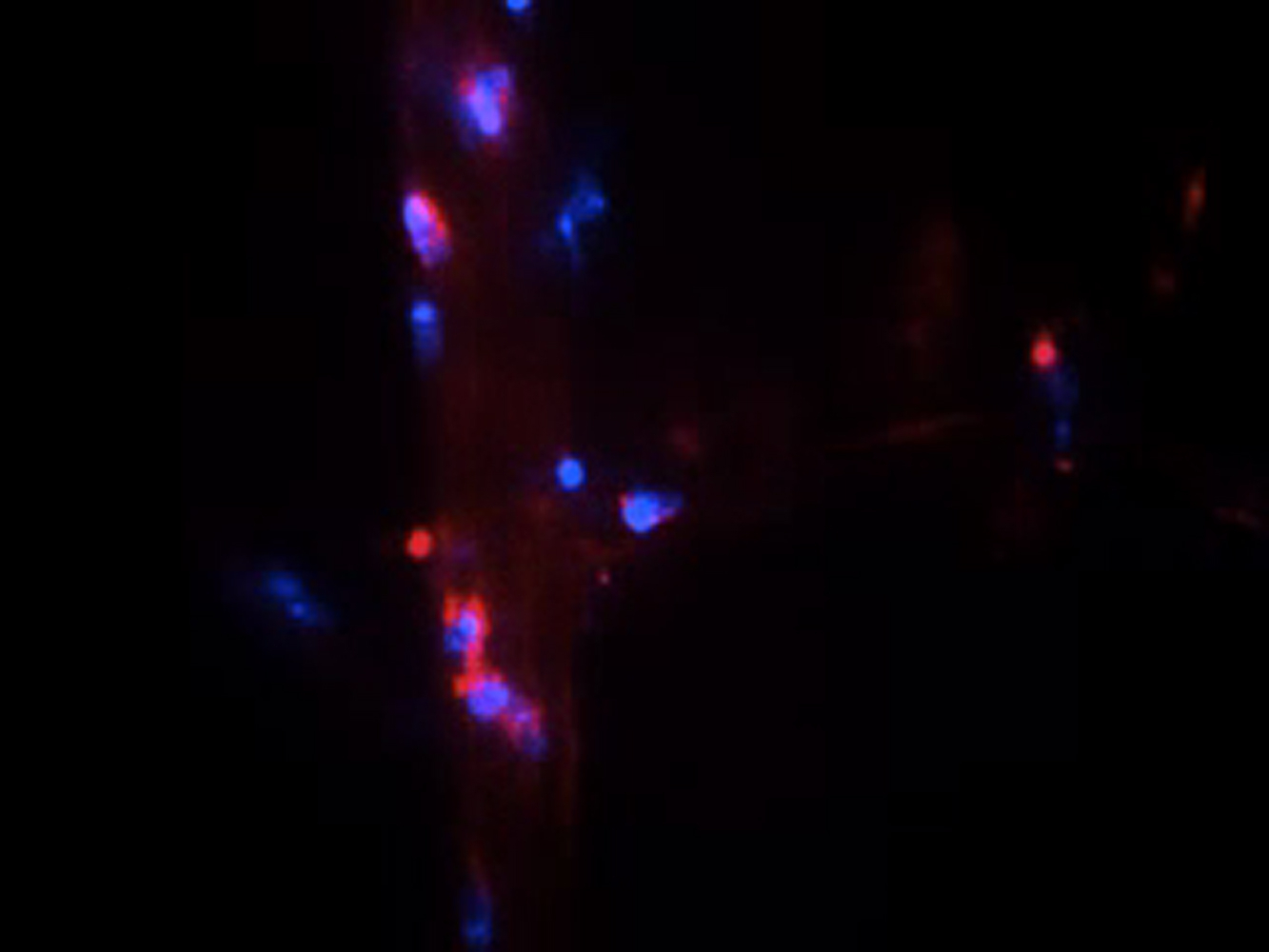

Supplement: Movie S2. Burn + Vehicle [file mmc3.jpg]

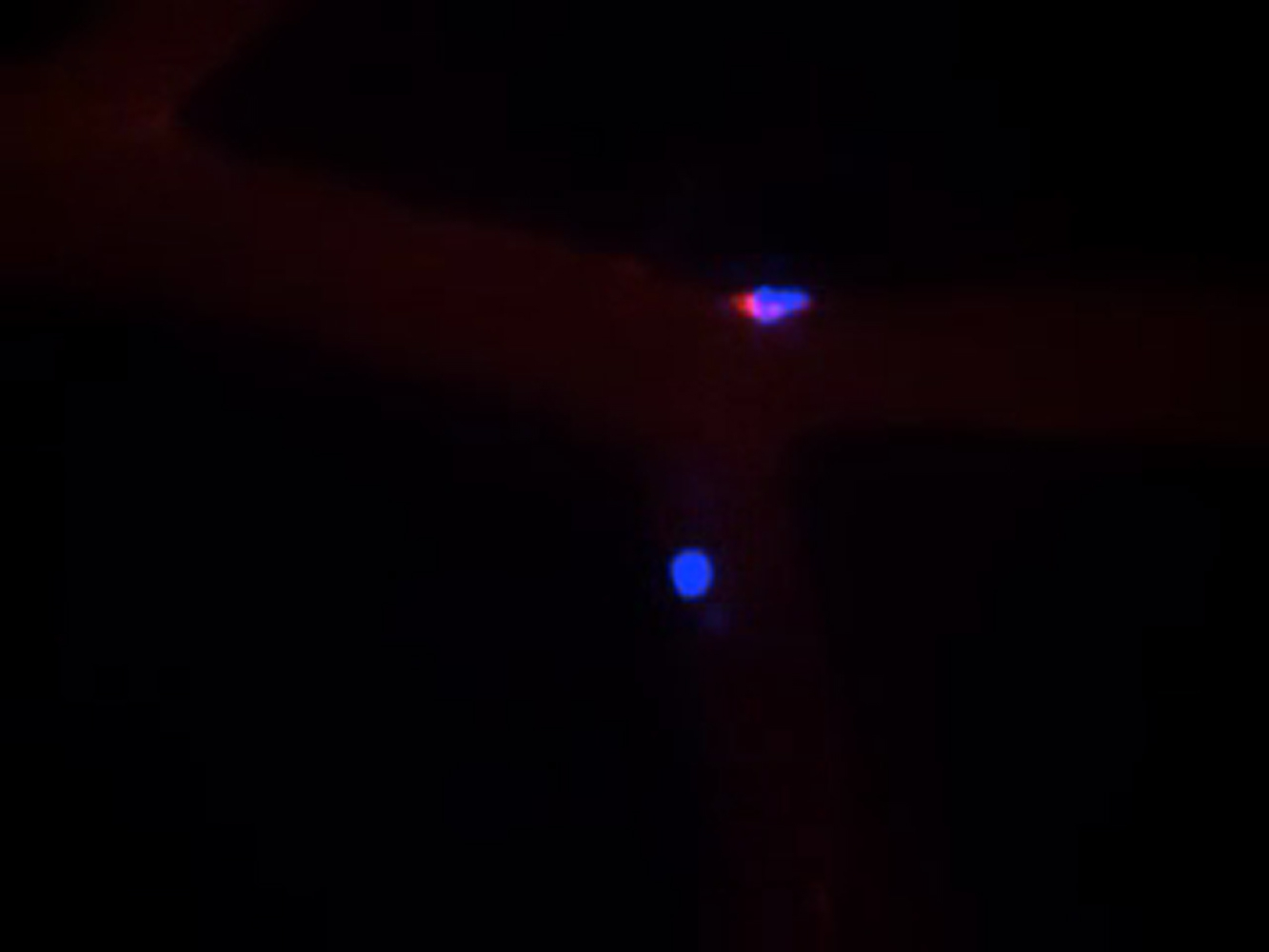

Supplement: Movie S3. Burn + C15 — High-power (20×) views of neutrophil (Ly6G, blue) and platelet (CD49b, red) behavior within dermal postcapillary venules 2 hr after wounding. A single 580 ± 39 μm2 focal injury was induced on the surface of a dorsal skin flap with a modified electrocautery device. C15 (100 pg/wound) or vehicle (saline) was administered intradermally immediately after wounding. For sham (vehicle control) experiments, mice were prepared for intravital microscopy and imaged identically to injured animals, but no injury was induced. See also Figure 2. [file mmc4.jpg]
