# Supplementary material for: Resolution Mediator Chemerin15 Reprograms the Wound Microenvironment to Promote Repair and Reduce Scarring
Source: Curr Biol. 2014 Jun 16;24(12):1406–14. doi: 10.1016/j.cub.2014.05.006 (PMC4064685; doi:10.1016/j.cub.2014.05.006)
Supplement: Document S3. Article plus Supplemental Information [file mmc6.pdf]

Current Biology 24, 1406–1414, June 16, 2014 ©2014 The Authors http://dx.doi.org/10.1016/j.cub.2014.05.006

Report

Resolution Mediator Chemerin15 Reprograms the Wound Microenvironment to Promote Repair and Reduce Scarring

Jenna L. Cash,[1,2,3,*] Mark D. Bass,[4] Jessica Campbell,[4] Matthew Barnes,[5] Paul Kubes,[3] and Paul Martin[1,4,*]
[1]School of Physiology & Pharmacology, Medical Sciences, University Walk, Bristol University, Bristol BS8 1TD, UK
[2]William Harvey Research Institute, Barts and the London School of Medicine and Dentistry, Charterhouse Square, London EC1M 6BQ, UK
[3]Calvin, Phoebe, and Joan Snyder Institute for Infection, Immunity, & Inflammation, University of Calgary, Calgary, AB T2N 4N1, Canada
[4]School of Biochemistry, Medical Sciences, University Walk, Bristol University, Bristol BS8 1TD, UK
[5]Takeda Cambridge Ltd., 418 Cambridge Science Park, Milton Road, Cambridge CB4 0PZ, UK

Summary

Disorders of cutaneous repair can cause disability or death given that skin functions as a protective barrier against the external environment. The inflammatory response triggered by tissue damage is thought to play both positive (e.g., pathogen-killing) and negative (e.g., scarring) roles in repair [1–3]. Inflammatory resolution mediators such as chemerin15 (C15) control the magnitude and duration of the inflammatory response; however, their role in wound repair and scarring is unknown [4–8]. Here, we show that the C15 precursor, chemerin, and its receptor, ChemR23, are both upregulated after skin damage and that the receptor is expressed by macrophages, neutrophils, and keratinocytes. Dynamic live-imaging studies of murine cutaneous wounds demonstrate that C15 delivery dampens the immediate intravascular inflammatory events, including platelet adhesion to neutrophils, an important event in driving leukocyte recruitment. C15 administration indirectly accelerates wound closure while altering fibroblast-mediated collagen deposition and alignment to reduce scarring. Macrophage recruitment is restricted to the immediate wound site rather than spilling extensively into the adjacent tissue as in control wounds, and macrophage phenotype in C15-treated wounds is skewed toward a less inflammatory phenotype with reduced iNOS, increased Arginase-1, and lower wound tumor necrosis factor α (TNF-α) expression. Modulation of inflammatory resolution pathways in acute and chronic wounds may therefore provide a novel therapeutic avenue to improve repair and reduce scarring.

Results

Repair of adult cutaneous wounds is a complex process that restores cellular structures and tissue layers but culminates in a scar consisting of linear bundles of collagen as opposed to the more randomly oriented collagen bundles found in normal skin. Wound repair in embryos involves very little inflammation and scar-free healing, suggesting that inflammatory cells are causally involved in scarring [1, 2, 9]. Resolution mediators function to dampen the inflammatory response and promote its resolution [5, 10]; however, their potential role in the context of dermal wound repair has, surprisingly, barely been investigated. Studies have shown that mice lacking the resolution mediator annexin A1 suffer delayed wound closure [11] and that proresolving mediators, including resolvin E1 (RvE1), promote corneal re-epithelialization and organ regeneration [12, 13].

The anti-inflammatory and proresolving peptide chemerin15 (C15) is a 15 aa peptide derived from cleavage of chemerin, which promotes phagocytosis of microbes and inhibits heart damage after myocardial infarction in a mouse model [6–8]. Full-length chemerin is a proinflammatory protein that can be cleaved to generate antimicrobial peptides and anti-inflammatory species [8, 14, 15]. C15 acts through the G-protein coupled receptor ChemR23, expressed on macrophages and neutrophils [6, 16]. ChemR23 is a multifunctional receptor that transduces the anti-inflammatory and proresolving effects of C-terminal chemerin peptides, including C15 and the lipid RvE1, as well as the proinflammatory effects of full-length chemerin [17–19]. The aim of this study was to determine what role C15 might play in regulating the wound inflammatory response and how this might influence subsequent skin repair.

Chemerin and ChemR23 Expression during Wound Healing

Our full-thickness 4 mm excisional wounding model involves the complete removal of the epidermis, dermis, and panniculus carnosus, which is firmly adherent to the base of the dermis. Healing commences after the formation of a fibrin clot that is invaded by granulation tissue and by the migration of an epidermal tongue along the interface between the granulation tissue and the clot (schematic in Figure 1A).

We found few chemerin$^+$ (C15 precursor) cells in unwounded skin, but their numbers increased profoundly in granulation tissue—they peaked at days 1 and 4 after wounding before declining to negligible numbers by day 7 (Figure 1B). Macrophages, but not neutrophils, were found to be chemerin$^+$ (Figure S1, available online). ChemR23 was upregulated 10-fold in granulation tissue by day 1 and remained high until day 7 (Figure 1B); it was expressed by wound macrophages, neutrophils, and keratinocytes, but not fibroblasts (Figure S1).

C15 Improves the Rate and Quality of Wound Repair

To investigate the potential effect of the chemerin peptide C15 on wound repair, we administered peptide (100 pg/wound) or vehicle control in Pluronic gel immediately after wounding. Macroscopic analysis showed that wound closure was markedly accelerated at all stages of repair in C15-treated wounds (Figures 1C and 1D; Figure S1) with a corresponding reduction in granulation tissue (Figure 1E). Maximal differences in wound areas were observed 4 days after wounding (C15: 36% ± 5% versus vehicle; 70% ± 6% of starting wound size; Figure 1D). We also noted that C15 treatment resulted in

*Correspondence: jenna.cash@bristol.ac.uk (J.L.C.), paul.martin@bristol.ac.uk (P.M.)
This is an open access article under the CC BY license (http://creativecommons.org/licenses/by/3.0/).

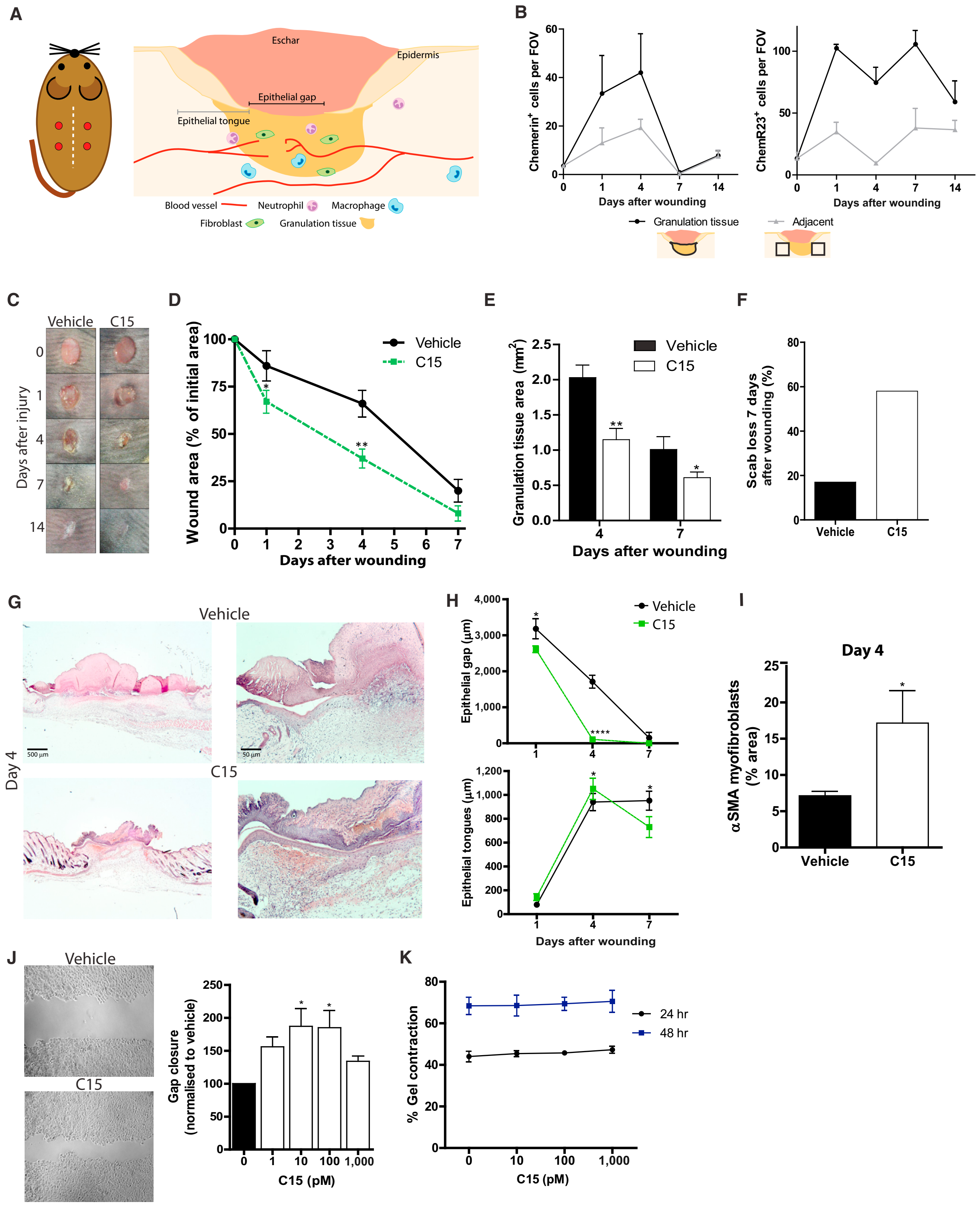

Figure 1. Skin Wound Healing Is Accelerated with C15 Treatment through Direct and Indirect Mechanisms

Four 4 mm excisional wounds were made to the dorsal skin of Sv129Ev mice. Vehicle or C15 (100 pg/wound) in 30% Pluronic gel was administered directly into the wound immediately after wounding.

(A) Schematic diagrams illustrating the location of skin wounds and measurements derived from histological sections.

(legend continued on next page)

significantly earlier scab loss—58% of wounds lost their scabs by day 7 in comparison to only 17% of vehicle-treated wounds (Figure 1F). These differences suggest that C15 promotes faster wound re-epithelialization, which may be directly by affecting keratinocyte migration and/or indirectly by modulating fibroblast behavior and leading to accelerated wound contraction. Indeed, epithelial gaps (schematic in Figure 1A) constituted only 4% of the width of day 4 C15-treated wounds in comparison to 60% of vehicle-treated wounds; however, epithelial tongues were only fractionally longer (Figures 1G and 1H), suggesting that quicker re-epithelialization is largely a consequence of a reduced denuded area available for re-epithelialization. Indeed, αSMA staining for contractile myofibroblasts (Figure 1I) indicated that day 4 C15-treated wounds possessed higher levels of these contractile cells than did control wounds. Interestingly, *ChemR23*$^{-/-}$ mice showed elevated neutrophil recruitment, but no significant changes in wound re-epithelialization or closure (Figure S2).

C15 Exerts Direct and Indirect Effects on Keratinocyte and Fibroblast Physiology

While our studies demonstrate that C15 can promote wound re-epithelialization and closure, it is unclear whether this is mediated entirely through C15's effects on inflammation or whether there might be some direct modulation of keratinocyte and fibroblast behavior by the peptide. We found that C15 modestly promoted keratinocyte migration into an in vitro "scratch wound" with optimal doses of 10–100 pM. (Figure 1J). However, C15 had no effect on fibroblast-mediated collagen gel contraction (Figure 1K). These data suggest that the accelerated wound closure observed in C15-treated wounds is via indirect mechanisms, whereas accelerated re-epithelialization could, in part, be through direct effects of C15 on keratinocytes.

C15 Regulates Neutrophil and Platelet Behavior Immediately after Wounding

Wound repair from the late stages of fetal development onward is accompanied by robust recruitment of inflammatory cells (neutrophils and monocytes) to the wound [20]. The optimal balance between sufficient leukocyte recruitment to combat any invading microorganisms and excessive pathological inflammation that contributes to scarring is clearly of critical importance in any tissue-repair response or therapeutic treatment [2, 20].

Platelets are essential for primary hemostasis, but they are also important amplifiers of acute inflammation. Activated platelets can trigger neutrophil recruitment by directly interacting with neutrophils to prompt a process called "secondary" neutrophil capture [21–23]. Platelets have previously been shown to express ChemR23 and respond to the ChemR23 lipid ligand RvE1 [24, 25], but the potential role of C15 in platelet biology has not been tested.

The standard excisional wound model described above is not amenable to live imaging of the dynamics of wound leukocyte and platelet behavior. To visualize the immediate inflammatory events occurring after cutaneous wounding, we used fluorescence spinning-disk intravital microscopy of small cutaneous burn wounds treated with vehicle or C15 (schematics in Figures 2A and 2B [26]).

At 2 hr after wounding, the first neutrophils were seen entering the wound, and the majority were found 150–500 μm from the edge of the burn. Even at this early stage, the effects of C15 were clear: we observed a 68% reduction in neutrophil numbers both within the wound and in the surrounding area (up to 500 μm; Figures 2C and 2D). Sham-operated animals exhibited low levels of neutrophil-endothelial cell interactions (Figures 2E and 2F), platelet-endothelial cell interactions (Figure 2G), and neutrophil-platelet interactions (Figure 2H). However, tissue injury elicited marked platelet and neutrophil rolling and adhesion within nearby postcapillary venules. Treatment with C15 significantly blunted these inflammatory responses. Specifically, 2 hr after burning, neutrophil adhesion was reduced by 74% (Figure 2F), platelet adhesion and rolling were inhibited by 76% and 62%, respectively (Figure 2G), and the percentage of neutrophils interacting with platelets dropped by 49% (Figure 2H; see also Movies S1, S2, and S3).

C15 Restricts Leukocyte Recruitment and Modulates Macrophage Phenotype during Repair

In the excisional wounding model, we monitored leukocyte recruitment dynamics during the repair period. C15 administration inhibited neutrophil recruitment to excisional cutaneous wounds by up to 60% on day 1 (50% on day 4; Figure 3A), but no changes in mast cell numbers were observed (Figure 3B). C15 reduced monocyte-macrophage recruitment on day 1 by 40%, but by day 7, there appeared to be no significant difference in the numbers of F4/80^{+} cells at the wound site (Figure 3C). However, more careful spatial analysis revealed a markedly different macrophage distribution in the immediately adjacent unwounded tissue (Figure 3D). Unlike vehicle-treated day 7 wounds, in which F4/80^{+} cells were abundant up to 3,000 μm from the wound edge, C15-treated wounds showed restricted monocyte-macrophage recruitment to the wound site such that macrophages extended only 356 ± 108 μm into the adjacent tissue (Figures 3D–3F). This suggests that C15 may function to prevent excessive

(B) Immunohistochemical staining during the wound repair time course revealed increases in the number of cells expressing chemerin and ChemR23 after wounding, particularly in granulation tissue.
(C) Macroscopic photos of vehicle- and C15-treated wounds 0–14 days after wounding.
(D) Wound area relative to initial wound area at days 1, 4, and 7 after wounding.
(E) Granulation tissue area at wound midpoints.
(F) Scab loss 7 days after wounding.
(G) Representative photos of sections from day 4 wound midpoints stained with haematoxylin and eosin.
(H) Re-epithelialization quantified by measurement of the length of wound epithelial gaps and tongues on days 1, 4, and 7 after wounding.
(I) αSMA myofibroblasts in day 4 wounds.
(J) HaCaTs (human keratinocytes) were allowed to migrate for 15 hr to fill in a "scratch wound" in vitro in the presence of C15 (1–1,000 pM) or media control.
(K) Murine dermal fibroblasts were suspended in a collagen I and media mixture supplemented with 10–1,000 pM C15 or vehicle (media) control. Gels were allowed to solidify at 37°C, and gel contraction was assessed 24 and 48 hr later.
Data are expressed as means ± SEM; there were six to ten mice (D and F) or four to eight wounds (B, E, H, and I) per treatment group or four independent experiments (J and K). *$p < 0.05$, **$p < 0.01$, and ***$p < 0.001$ relative to vehicle-treated controls. See also Figure S1. The following abbreviation is used: FOV, field of view.

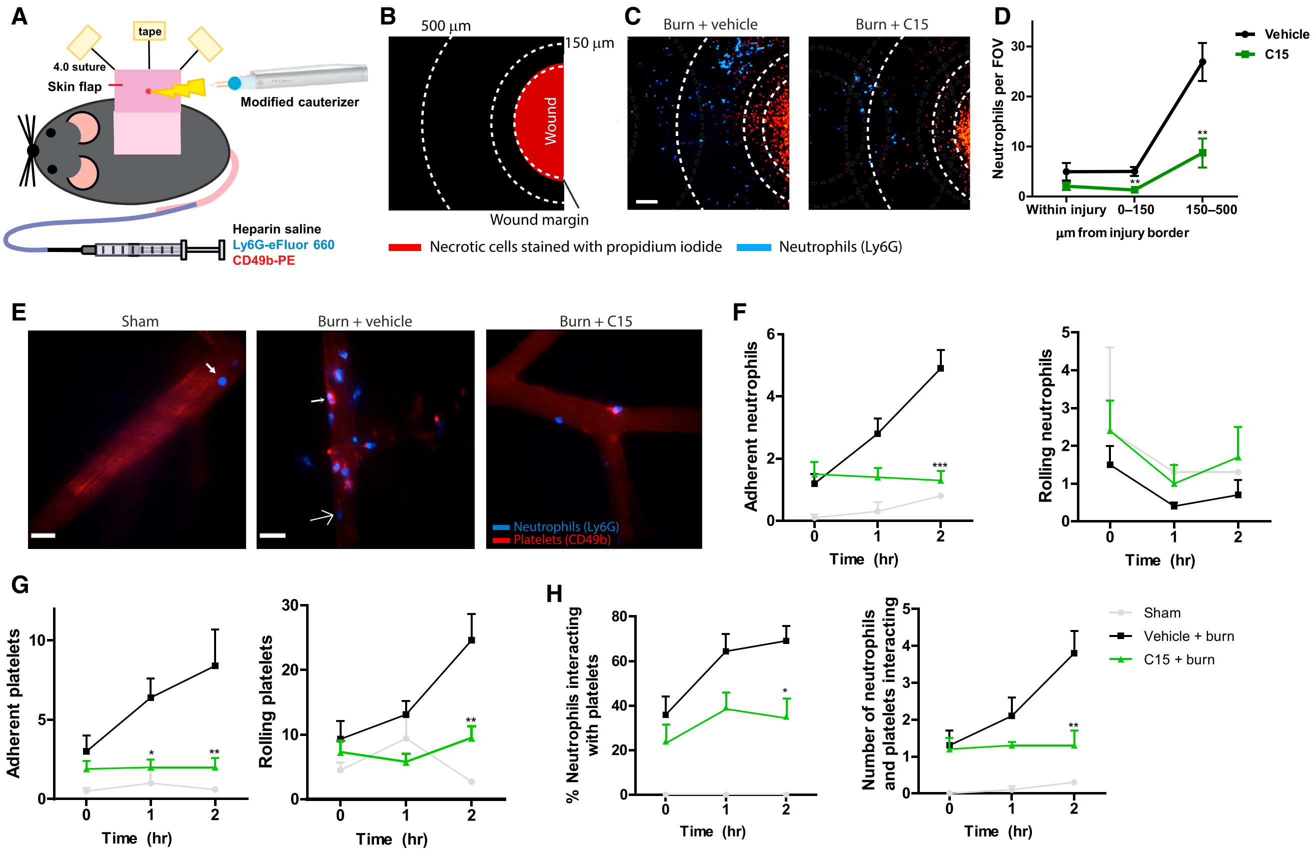

Figure 2. C15 Inhibits Early Platelet and Neutrophil Inflammatory Events after Cutaneous Wounding

A single 580 ± 39 μm^2 focal injury was induced on the surface of a dorsal skin flap with a modified electrocautery device. C15 (100 pg/wound) or vehicle (saline) was administered intradermally immediately after wounding. For sham experiments, mice were prepared for intravital microscopy and imaged identically to injured animals, but no injury was induced.

(A) Schematic diagram of the skin preparation with burn injury for multichannel fluorescence spinning-disk confocal microscopy.

(B) Schematic showing the wound as viewed with a 4× objective; necrotic cells stained with propidium iodide are shown in red.

(C) Low-power (4×) views of necrotic cells (propidium iodide, red) and neutrophil recruitment (blue) to vehicle- or C15-treated wounds 2 hr after injury. The scale bar indicates 150 μm. Dotted lines indicate the wound margin, 150 μm and 500 μm from the wound edge.

(D) Quantification of neutrophil recruitment to wounds treated with vehicle or C15.

(E) High-power (20x) views of neutrophil (Ly6G, blue) and platelet (CD49b, red) behavior within dermal postcapillary venules 2 hr after wounding. The scale bar indicates 30 μm. The arrow in “Sham” indicates a rolling neutrophil. The large arrow in “Burn + vehicle” indicates a neutrophil without interacting platelets. The small arrow indicates neutrophils with interacting platelets.

(F–H) Quantification of neutrophil rolling and adhesion (F), platelet rolling and adhesion (G), and platelet-neutrophil interactions (H) after wounding.

Data are expressed as means ± SEM; four to eight vessels were visualized per time point in each mouse, and there were four to eight animals per treatment group. Vehicle (n = 4), burn (n = 8), C15 (n = 7). *$p < 0.05$, **$p < 0.01$, and ***$p < 0.001$ relative to vehicle-treated mice. See also Movies S1, S2, and S3. The following abbreviation is used: FOV, field of view.

macrophage recruitment, which could be detrimental to the wound repair process, and to direct macrophages to leave the wound earlier. Indeed, by day 14, markedly fewer F4/80$^+$ cells were detectable at the wound site. This may be of significant potential therapeutic benefit since spreading of the inflammatory response beyond the immediate wound site may lead to excessive scarring, as seen in extreme cases with keloid scars [27].

We also noted that a high proportion of vehicle-treated wound macrophages exhibited a round morphology, whereas C15-treated wound macrophages had many protrusions, which may reflect a more migratory or phagocytic phenotype (Figure 3F). Indeed, C15-treated wounds exhibited higher levels of Prussian blue$^+$ iron-loaded cells (macrophages with phagocytosed erythrocytes) at days 4 and 7 after wounding, and these cells appeared to track away from the wound (Figure 3G). Increased wound haem clearance could aid repair by removing a known stimulus of “M1” macrophage activation, which has previously been associated with poor repair in iron-burdened chronic wounds [28]. Analysis of tumor necrosis factor α (TNF-α) expression revealed markedly lower levels within the wound granulation tissue after C15 treatment, suggestive of a less inflamed environment (Figure 3H). Moreover, phenotypic analysis of wound macrophages showed that more macrophages in C15-treated wounds expressed Arginase-1, typically associated with an “M2” phenotype, and fewer expressed iNOS, typically associated with an “M1” phenotype, although no change in the “M2 marker” Ym1 was observed (Figure 3I).

Collectively, these data indicate that C15 administration not only acts to restrict inflammatory cell recruitment to the wound site but may also direct earlier resolution and skew macrophage phenotype toward that typically linked with improved wound repair.

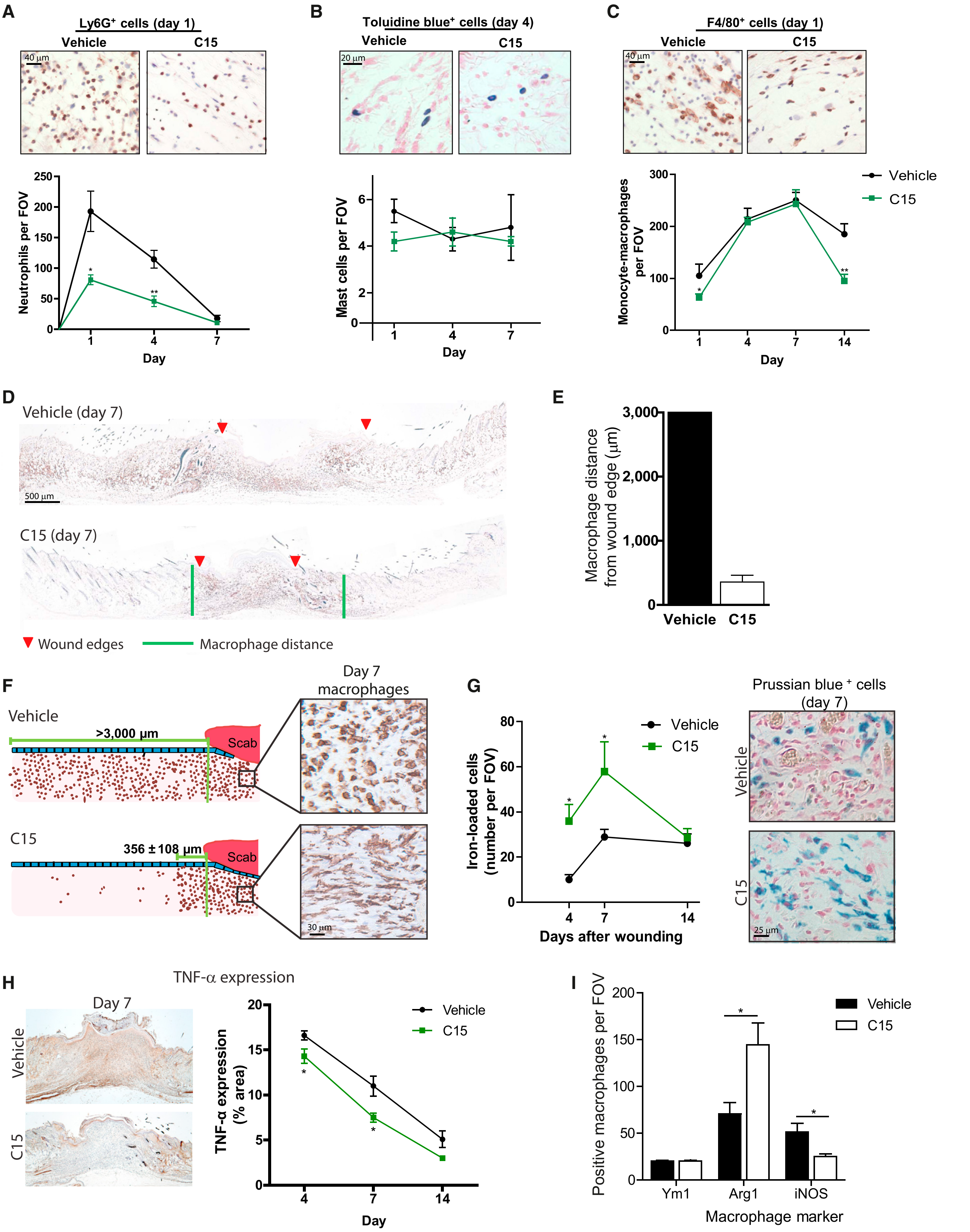

Figure 3. C15 Dampens Wound Leukocyte Recruitment and Skews Macrophage Phenotype

Four 4 mm excisional wounds were made to the dorsal skin of Sv129Ev mice. Vehicle or C15 (100 pg/wound) in 30% Pluronic gel was administered directly into the wound immediately after wounding.

(A–C) Representative micrographs and quantification of neutrophil (Ly6G$^+$ cell, brown) (A), mast cell (toluidine blue$^+$ cell, blue) (B), and monocyte-macrophage (F4/80$^+$ cell, brown) (C) recruitment to wound granulation tissue up to 14 days after wounding.

(legend continued on next page)

C15 Inhibits Scarring by Modifying Collagen Organization within the Healing Wound

Collagen is a major structural component of normal skin, and prior to wounding, it exhibits a basketweave appearance in the dermis, whereas after healing, collagen is thought to be deposited in aligned bundles characteristic of a scar. The degree of scarring has previously been assessed either by measurement of collagen levels within the wound or by subjective measurement of collagen fiber orientation [29, 30].

To better assess collagen fiber orientation, we designed a novel algorithm to quantify collagen fiber alignment within wounded versus unwounded dermis (Figure S3). As expected, unwounded skin displayed abundant collagen with a basketweave appearance (largely randomly oriented fibers) with a low alignment (0.57 ± 0.003; Figures 4A–4C). In vehicle-treated day 7 wounds, collagen was laid down in a more linear fashion, resulting in a high alignment of 0.90 ± 0.01. Wound treatment with C15, however, resulted in collagen deposition with a more random collagen fiber orientation closer to unwounded dermal collagen organization, which equated to reduced alignment in comparison to that of vehicle-treated wounded skin (C15: 0.71 ± 0.02; Figures 4A–4C). The data obtained from our alignment algorithm compare favorably with those of an established technique for assessing collagen quantity by viewing wounds stained with picrosirius red under polarized light (Figure S3) and suggest that a downstream consequence of C15 treatment appears to be a reduction in the degree of scarring at repair sites.

Discussion

Various genetic and pharmacological approaches for knocking down the wound inflammatory response have suggested that leukocytes deliver signals that lead to scarring [2, 31]. Clearly, genetic deletion of immune cells is not a practical clinical option to control scarring; however, since resolution mediators drive the cessation of the inflammatory response, they may provide therapeutic targets for modulating wound inflammation in an attempt to regulate the repair and scarring process [10, 32].

In this study, we assessed the impact of a proresolving mediator on dermal wound repair and scarring. By administering the anti-inflammatory and proresolving peptide C15 to wounds, we were able to speed up wound closure, re-epithelialization, and scab loss while suppressing wound neutrophil and macrophage recruitment and limiting scarring. C15 most likely achieves these effects through direct modulation of leukocyte dynamics at the wound site since our previous studies have demonstrated that C15 can suppress activation of integrins required to mediate neutrophil adhesion and transendothelial migration [6]. This may, in part, be the mechanism underpinning our observed reduction in neutrophil recruitment to excisional wounds (Figure 3A) and dampened neutrophil-endothelial and neutrophil-platelet interactions at burn lesions (Figure 2). Similar observations have been made by others with the resolution mediator resolvin D2 [33]. Furthermore, C15 stimulates a switch of macrophage phenotype to cells expressing M2-like markers, including arginase 1, and reduced expression of M1 markers, including iNOS and TNF-α. The resolution mediators RvE1 and maresin have also recently been shown to be capable of switching macrophage phenotype, suggesting that a defining characteristic of proresolving mediators may be their ability to switch macrophage phenotype [34].

C15 does not promote wound closure by directly regulating fibroblast physiology but rather does so through indirect actions. We postulate that leukocytes exposed to C15 in the wound bed signal to fibroblasts either through a soluble mediator or via cell-cell contacts to promote conversion to contractile myofibroblasts and thus faster contraction of the wound.

In addition, we suggest a role for C15 in regulating platelet behavior at the site of tissue damage. Platelets play a plethora of roles in physiology, but a definitive role in wound repair has yet to be established. It is known that platelets drive wound neutrophil and macrophage recruitment and secrete transforming growth factor β (TGF-β), and thus it would be tempting to assume that since wound leukocyte recruitment and TGF-β drive scarring, platelets, by proxy, might contribute to scarring. Our live-imaging studies indicate that one step where this might be driven is by platelet “capturing” of neutrophils in vessels near the wound and that C15 treatment inhibits this process.

Although it has been known for some time that collagen in scars exhibits a linear, bundled arrangement while unwounded skin exhibits a looser, basketweave appearance, very little is known about the dynamics of collagen deposition in wounds and how the behavior of fibroblasts is altered to lead to these very different patterns of collagen deposition. What is clear is that signals from inflammatory cells at the wound site directly influence these fibroblast behaviors, and thus by modulating the wound inflammatory response, it is possible to dramatically modulate the scarring phenotype. This, to a large degree, is what we have achieved through application of C15. A next step will clearly be to investigate further how immune cells change fibroblast behaviors because this will provide further therapeutic targets for blocking scarring downstream of immune regulators.

Although the cellular and molecular mechanisms behind repair and scar formation have yet to be fully elucidated, this study suggests that targeting the body’s endogenous resolution mechanisms could provide a potential therapeutic route to promote repair and minimize scarring through modulation of leukocyte and stromal cell physiology.

Experimental Procedures

All experiments were conducted with approval from the local ethical review committee at the University of Bristol and in accordance with the UK Home Office regulations (Guidance on the Operation of Animals, Scientific

(D) Macrophage immunostaining through the midpoint of day 7 wounds. Red arrows indicate wound edges, and green lines mark the edge of the macrophage-dense area.
(E) Quantification of the distance that macrophages extend beyond the wound margin into the surrounding tissue.
(F) Schematic showing macrophage distribution around vehicle- and C15-treated day 7 wounds and corresponding photos of macrophage morphology.
(G) Quantification and representative photos of Prussian blue$^+$ iron-loaded cells within the wound.
(H) Quantification and representative photos of TNF-α expression (brown).
(I) Characterization of macrophage phenotype within day 7 C15- and vehicle-treated wounds. Ym1 and Arg1 are M2 markers, and iNOS is an M1 macrophage marker.
Data are expressed as means ± SEM; there were four to eight wounds per treatment group. *$p < 0.05$ and **$p < 0.01$ relative to vehicle-treated wounds. The following abbreviation is used: FOV, field of view.

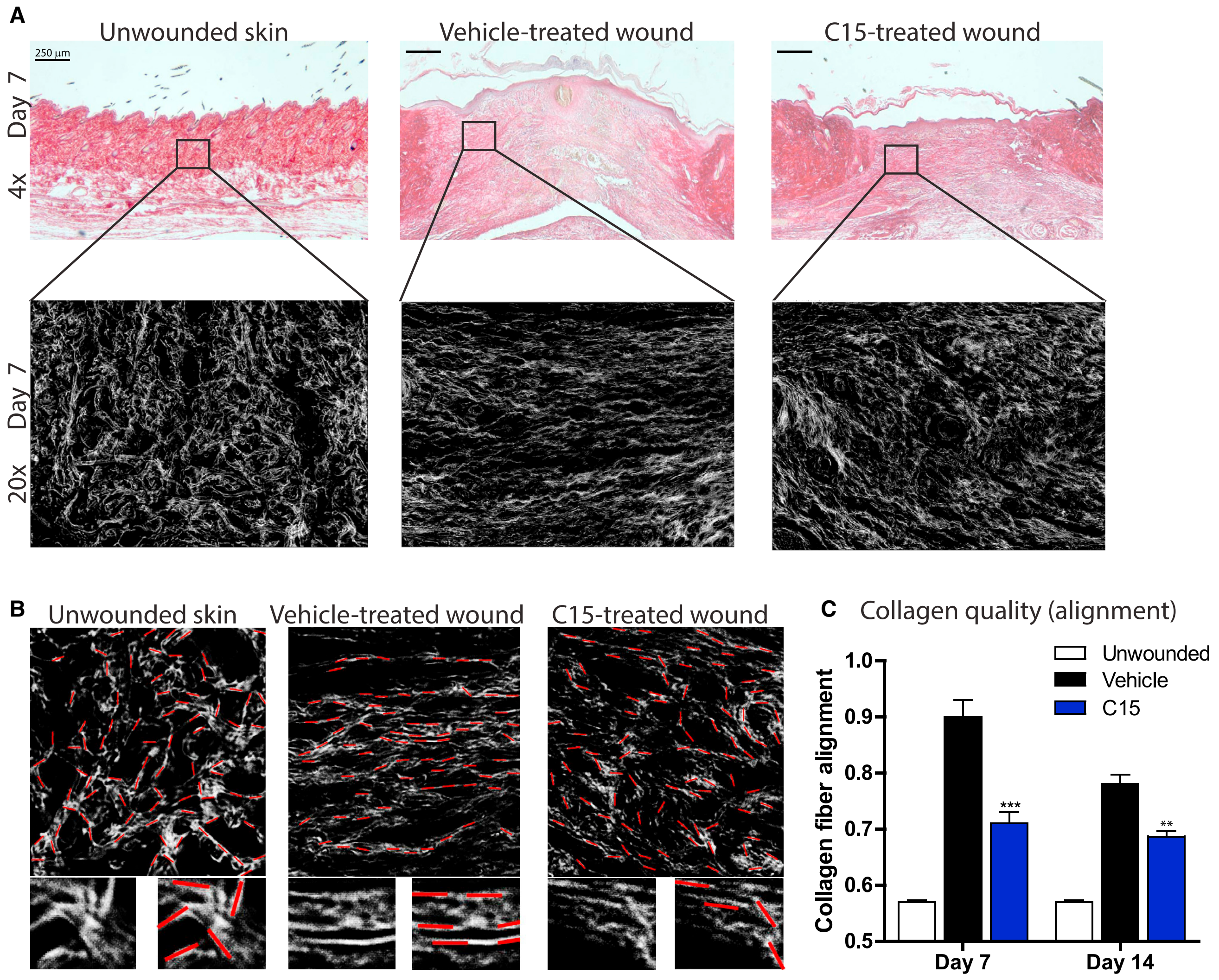

Figure 4. C15 Inhibits Scar Formation

(A) Day 7 wound midsections (4× objective on top; 20× objective on bottom) stained with picrosirius red (collagen) from unwounded skin and vehicle- and C15-treated wounds.

(B) Images used for algorithm analysis (zoomed-in areas are shown underneath). Red lines were fitted by the algorithm along white lines of collagen, and the angles of each line were measured and compared for determining fiber alignment.

(C) Quantification of collagen fiber alignment in day 7 and 14 wounds. An entirely randomly oriented network of collagen fibers would score 0.5 in this analysis, whereas complete fiber alignment would score 1.

Data are expressed as means ± SEM; there were six wounds per treatment group. ***$p < 0.001$ and **$p < 0.01$ relative to vehicle-treated wounds. See also Figure S3 and Supplemental Experimental Procedures.

Procedures Act, 1986). Wild-type (Sv129Ev) and *ChemR23*$^{-/-}$ mice were supplied by Takeda Pharmaceuticals and used for excisional wounding studies. C57Bl6/J mice were purchased from Jackson Laboratories (Canada) and used for intravital imaging experiments.

C15

C15 (AGEDPHGYFLPGQFA) was synthesized by Mimotopes, reconstituted (1 mM in PBS and 0.1% BSA), and stored at −80°C for up to 6 months. Peptide was diluted from fresh aliquots for each experiment.

Excisional Cutaneous Wounding

Mice (7–10 weeks old) were randomly assigned a treatment group and anaesthetized with isofluorane. Four full-thickness excisional wounds (4 mm biopsy punch, Kai Industries) were made to the shaved dorsal skin. Vehicle (PBS) or C15 (100 pg/wound) was delivered topically by pipette into the wound cavity immediately after wounding (40 µl in a vehicle of 30% Pluronic F-127 gel [is liquid at 4°C but solidifies at body temperature]; Sigma Aldrich). Wounds were photographed with an Olympus camera and calibration card on days 0, 1, 4, 7, and 14 after wounding, and wound areas were calculated with ImageJ software.

Preparation and Histological Analysis of Excisional Cutaneous Wounds

Wounds were harvested, prepared, and analyzed essentially as previously described [35] (for detailed methods, see the Supplemental Experimental Procedures). Because of the lack of a commercially available anti-ChemR23 antibody suitable for formalin-fixed, paraffin-embedded immunohistochemistry, we immunostained for β-galactosidase (2 µg/ml, Invitrogen) in *Cmklr1*$^{-/-}$ LacZ reporter mice to determine the ChemR23 expression profile.

Intravital Imaging of Cutaneous Focal Necrotic Injury

Skin preparation for intravital microscopy was performed essentially as previously described [26] (for further detail, see the Supplemental Experimental Procedures)—C15 or vehicle control was administered intradermally immediately after wounding.

Collagen Alignment

We designed an algorithm to quantify the level of collagen fiber alignment within tissue sections stained with picrosirius red. We used 2D fast Fourier transformations to scan images for nonrandom, noncircular regions and to measure the angle of fitted ellipses. The algorithm quantified the alignment of fibers by comparing each angle with every other from an image such that we gave parallel fibers a score of 1 and perpendicular fibers a score of 0. Two perpendicular lines have an alignment of 0; therefore, in a random arrangement, total alignment = 0.5, and in perfectly aligned tissue, alignment = 1. We performed the analysis by using our novel algorithm in ImageJ software and Silverlight software. For further details, see the Supplemental Experimental Procedures and Figure S3.

Statistical Analysis

Student's t test and one- and two-way ANOVA with the Bonferroni post hoc test were performed with GraphPad Prism 5.0 software.

Supplemental Information

Supplemental Information includes three figures, Supplemental Experimental Procedures, three movies, and two Supplemental Algorithms and can be found with this article online at http://dx.doi.org/10.1016/j.cub.2014.05.006.

Author Contributions

J.L.C. conceived and planned the project, designed and performed the experiments, analyzed data, and wrote the manuscript. M.D.B. wrote software and analyzed data. J.C. performed experiments. M.B. provided wild-type and *ChemR23*$^{-/-}$ mice. P.K. contributed to the writing of the manuscript. P.M. planned the project and wrote the manuscript.

Acknowledgments

This work was funded by a Sir Henry Wellcome Postdoctoral Fellowship (088967/Z/09/Z) awarded to J.L.C., a Wellcome Investigator Award to P.M., a Wellcome Career Development Fellowship to M.D.B., and a Wellcome PhD studentship to J.C. We thank Debbie Martin and Debi Ford for histological services, Rob Nunan, Connie Wong, Bjoern Petri, and Lori Zybutnik for advice, and Josie Morris for providing fibroblasts.

Received: December 9, 2013
Revised: March 26, 2014
Accepted: May 2, 2014
Published: May 29, 2014

References

1. Martin, P., D'Souza, D., Martin, J., Grose, R., Cooper, L., Maki, R., and McKercher, S.R. (2003). Wound healing in the PU.1 null mouse—tissue repair is not dependent on inflammatory cells. Curr. Biol. *13*, 1122–1128.
2. Stramer, B.M., Mori, R., and Martin, P. (2007). The inflammation-fibrosis link? A Jekyll and Hyde role for blood cells during wound repair. J. Invest. Dermatol. *127*, 1009–1017.
3. Silva, M.T. (2010). When two is better than one: macrophages and neutrophils work in concert in innate immunity as complementary and cooperative partners of a myeloid phagocyte system. J. Leukoc. Biol. *87*, 93–106.
4. Gilroy, D.W., Lawrence, T., Perretti, M., and Rossi, A.G. (2004). Inflammatory resolution: new opportunities for drug discovery. Nat. Rev. Drug Discov. *3*, 401–416.
5. Serhan, C.N., Brain, S.D., Buckley, C.D., Gilroy, D.W., Haslett, C., O'Neill, L.A., Perretti, M., Rossi, A.G., and Wallace, J.L. (2007). Resolution of inflammation: state of the art, definitions and terms. FASEB J. *21*, 325–332.
6. Cash, J.L., Bena, S., Headland, S.E., McArthur, S., Brancaleone, V., and Perretti, M. (2013). Chemerin15 inhibits neutrophil-mediated vascular inflammation and myocardial ischemia-reperfusion injury through ChemR23. EMBO Rep. *14*, 999–1007.
7. Cash, J.L., Christian, A.R., and Greaves, D.R. (2010). Chemerin peptides promote phagocytosis in a ChemR23- and Syk-dependent manner. J. Immunol. *184*, 5315–5324.
8. Cash, J.L., Hart, R., Russ, A., Dixon, J.P., Colledge, W.H., Doran, J., Hendrick, A.G., Carlton, M.B., and Greaves, D.R. (2008). Synthetic chemerin-derived peptides suppress inflammation through ChemR23. J. Exp. Med. *205*, 767–775.
9. Hopkinson-Woolley, J., Hughes, D., Gordon, S., and Martin, P. (1994). Macrophage recruitment during limb development and wound healing in the embryonic and foetal mouse. J. Cell Sci. *107*, 1159–1167.
10. Serhan, C.N., Chiang, N., and Van Dyke, T.E. (2008). Resolving inflammation: dual anti-inflammatory and pro-resolution lipid mediators. Nat. Rev. Immunol. *8*, 349–361.
11. Norling, L.V., Spite, M., Yang, R., Flower, R.J., Perretti, M., and Serhan, C.N. (2011). Cutting edge: Humanized nano-proresolving medicines mimic inflammation-resolution and enhance wound healing. J. Immunol. *186*, 5543–5547.
12. Serhan, C.N., Dalli, J., Karamnov, S., Choi, A., Park, C.K., Xu, Z.Z., Ji, R.R., Zhu, M., and Petasis, N.A. (2012). Macrophage proresolving mediator maresin 1 stimulates tissue regeneration and controls pain. FASEB J. *26*, 1755–1765.
13. Biteman, B., Hassan, I.R., Walker, E., Leedom, A.J., Dunn, M., Seta, F., Laniado-Schwartzman, M., and Gronert, K. (2007). Interdependence of lipoxin A4 and heme-oxygenase in counter-regulating inflammation during corneal wound healing. FASEB J. *21*, 2257–2266.
14. Kulig, P., Kantyka, T., Zabel, B.A., Banas, M., Chyra, A., Stefanska, A., Tu, H., Allen, S.J., Handel, T.M., Kozik, A., et al. (2011). Regulation of chemerin chemoattractant and antibacterial activity by human cysteine cathepsins. J. Immunol. *187*, 1403–1410.
15. Banas, M., Zabieglo, K., Kasetty, G., Kapinska-Mrowiecka, M., Borowczyk, J., Drukala, J., Murzyn, K., Zabel, B.A., Butcher, E.C., Schroeder, J.M., et al. (2013). Chemerin is an antimicrobial agent in human epidermis. PLoS ONE *8*, e58709.
16. Samson, M., Edinger, A.L., Stordeur, P., Rucker, J., Verhasselt, V., Sharron, M., Govaerts, C., Mollereau, C., Vassart, G., Doms, R.W., and Parmentier, M. (1998). ChemR23, a putative chemoattractant receptor, is expressed in monocyte-derived dendritic cells and macrophages and is a coreceptor for SIV and some primary HIV-1 strains. Eur. J. Immunol. *28*, 1689–1700.
17. Arita, M., Ohira, T., Sun, Y.P., Elangovan, S., Chiang, N., and Serhan, C.N. (2007). Resolvin E1 selectively interacts with leukotriene B4 receptor BLT1 and ChemR23 to regulate inflammation. J. Immunol. *178*, 3912–3917.
18. Arita, M., Yoshida, M., Hong, S., Tjonahen, E., Glickman, J.N., Petasis, N.A., Blumberg, R.S., and Serhan, C.N. (2005). Resolvin E1, an endogenous lipid mediator derived from omega-3 eicosapentaenoic acid, protects against 2,4,6-trinitrobenzene sulfonic acid-induced colitis. Proc. Natl. Acad. Sci. USA *102*, 7671–7676.
19. Yoshimura, T., and Oppenheim, J.J. (2011). Chemokine-like receptor 1 (CMKLR1) and chemokine (C-C motif) receptor-like 2 (CCRL2); two multifunctional receptors with unusual properties. Exp. Cell Res. *317*, 674–684.
20. Eming, S.A., Krieg, T., and Davidson, J.M. (2007). Inflammation in wound repair: molecular and cellular mechanisms. J. Invest. Dermatol. *127*, 514–525.
21. Zarbock, A., Polanowska-Grabowska, R.K., and Ley, K. (2007). Platelet-neutrophil-interactions: linking hemostasis and inflammation. Blood Rev. *21*, 99–111.
22. Martin, P., and Leibovich, S.J. (2005). Inflammatory cells during wound repair: the good, the bad and the ugly. Trends Cell Biol. *15*, 599–607.
23. Nurden, A.T. (2011). Platelets, inflammation and tissue regeneration. Thromb. Haemost. *105* (*Suppl 1*), S13–S33.
24. Dona, M., Fredman, G., Schwab, J.M., Chiang, N., Arita, M., Goodarzi, A., Cheng, G., von Andrian, U.H., and Serhan, C.N. (2008). Resolvin E1, an EPA-derived mediator in whole blood, selectively counterregulates leukocytes and platelets. Blood *112*, 848–855.
25. Fredman, G., Van Dyke, T.E., and Serhan, C.N. (2010). Resolvin E1 regulates adenosine diphosphate activation of human platelets. Arterioscler. Thromb. Vasc. Biol. *30*, 2005–2013.
26. McDonald, B., Pittman, K., Menezes, G.B., Hirota, S.A., Slaba, I., Waterhouse, C.C., Beck, P.L., Muruve, D.A., and Kubes, P. (2010). Intravascular danger signals guide neutrophils to sites of sterile inflammation. Science *330*, 362–366.
27. Shih, B., Garside, E., McGrouther, D.A., and Bayat, A. (2010). Molecular dissection of abnormal wound healing processes resulting in keloid disease. Wound Repair Regen. *18*, 139–153.
28. Sindrilaru, A., Peters, T., Wieschalka, S., Baican, C., Baican, A., Peter, H., Hainzl, A., Schatz, S., Qi, Y., Schlecht, A., et al. (2011). An unrestrained proinflammatory M1 macrophage population induced by

iron impairs wound healing in humans and mice. J. Clin. Invest. *121*, 985–997.
29. Ferguson, M.W., Duncan, J., Bond, J., Bush, J., Durani, P., So, K., Taylor, L., Chantrey, J., Mason, T., James, G., et al. (2009). Prophylactic administration of avotermin for improvement of skin scarring: three double-blind, placebo-controlled, phase I/II studies. Lancet *373*, 1264–1274.
30. Menon, S.N., Flegg, J.A., McCue, S.W., Schugart, R.C., Dawson, R.A., and McElwain, D.L. (2012). Modelling the interaction of keratinocytes and fibroblasts during normal and abnormal wound healing processes. Proc. Biol. Sci. *279*, 3329–3338.
31. Eming, S.A., Hammerschmidt, M., Krieg, T., and Roers, A. (2009). Interrelation of immunity and tissue repair or regeneration. Semin. Cell Dev. Biol. *20*, 517–527.
32. Lawrence, T., Willoughby, D.A., and Gilroy, D.W. (2002). Anti-inflammatory lipid mediators and insights into the resolution of inflammation. Nat. Rev. Immunol. *2*, 787–795.
33. Bohr, S., Patel, S.J., Sarin, D., Irimia, D., Yarmush, M.L., and Berthiaume, F. (2013). Resolvin D2 prevents secondary thrombosis and necrosis in a mouse burn wound model. Wound Repair Regen. *21*, 35–43.
34. Dalli, J., Zhu, M., Vlasenko, N.A., Deng, B., Haeggström, J.Z., Petasis, N.A., and Serhan, C.N. (2013). The novel 13S,14S-epoxy-maresin is converted by human macrophages to maresin 1 (MaR1), inhibits leukotriene A4 hydrolase (LTA4H), and shifts macrophage phenotype. FASEB J. *27*, 2573–2583.
35. Mori, R., Shaw, T.J., and Martin, P. (2008). Molecular mechanisms linking wound inflammation and fibrosis: knockdown of osteopontin leads to rapid repair and reduced scarring. J. Exp. Med. *205*, 43–51.

Current Biology, Volume *24*

Supplemental Information

Resolution Mediator Chemerin15

Reprograms the Wound Microenvironment

to Promote Repair and Reduce Scarring

Jenna L. Cash, Mark D. Bass, Jessica Campbell, Matthew Barnes, Paul Kubes, and Paul Martin

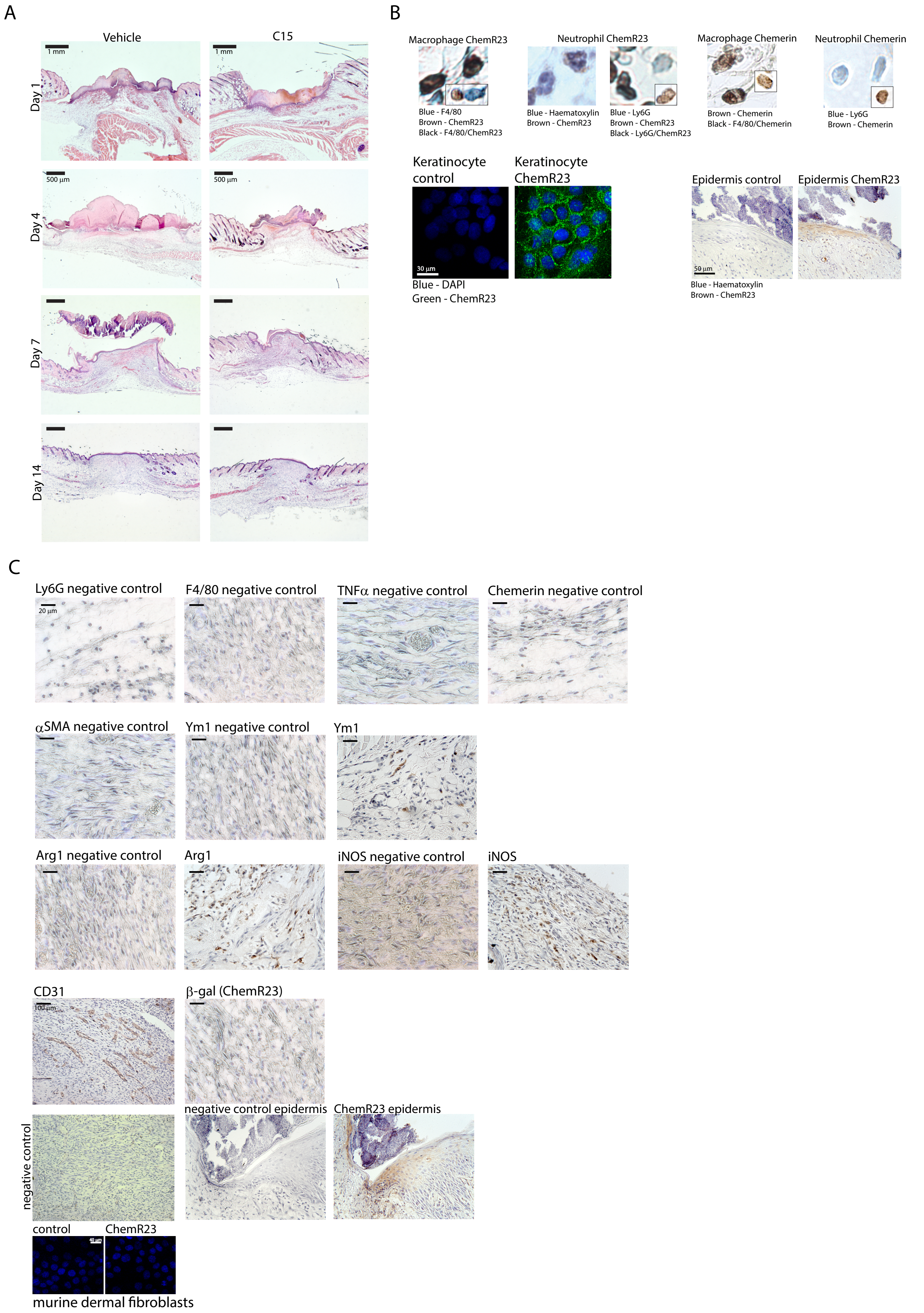

Figure S1. A Low magnification views of H&E stained wounds throughout the repair time course.
B Immunohistochemical and immunofluoresence staining to identify cell lineages expressing chemerin and ChemR23. Macrophages (F4/80+, blue), neutrophils (Ly6G and haematoxylin, blue), double staining (black/dark brown), chemerin and ChemR23 (brown). Boxes in the right hand corner of the images highlight examples of single stained cells.
C Negative controls for immunohistochemical and immunofluoresence stains. DAPI, blue. ChemR23 (Alexa 488, green or DAB, brown). See Fig.1

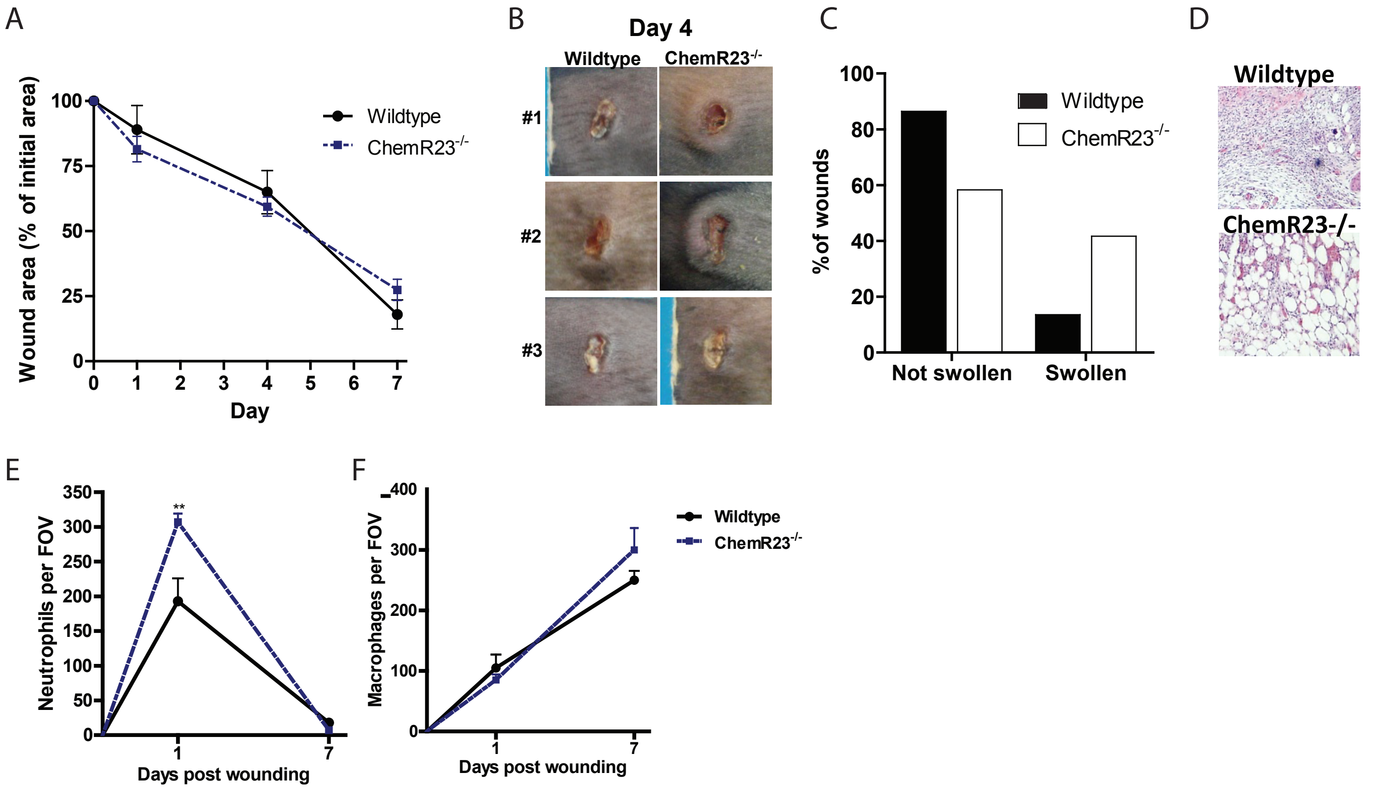

Figure S2. Wound repair in ChemR23-/- mice

A Wound area relative to initial wound area at days 1, 4 and 7 post-wounding. **B** Macroscopic photos of wildtype and ChemR23-/- wounds at day 4 post-wounding. **C** Percentage of swollen wounds in wildtype and ChemR23-/- mice 4 days post-wounding. **D** Representative haematoxylin and eosin stained wound mid-sections from day 4 wounds. **E** Neutrophil (Ly6G+ cell) and **F** macrophage (F4/80+ cell) recruitment to wound granulation tissue up to 7 days post-wounding. Data are expressed as means +/- SEM with 6 mice per experimental group (**A,C**) and 4-6 wounds per group analysed for neutrophil and macrophage recruitment. (**E,F**). **, $p<0.01$ relative to wildtype wounds. FOV, field of view. See Fig.3.

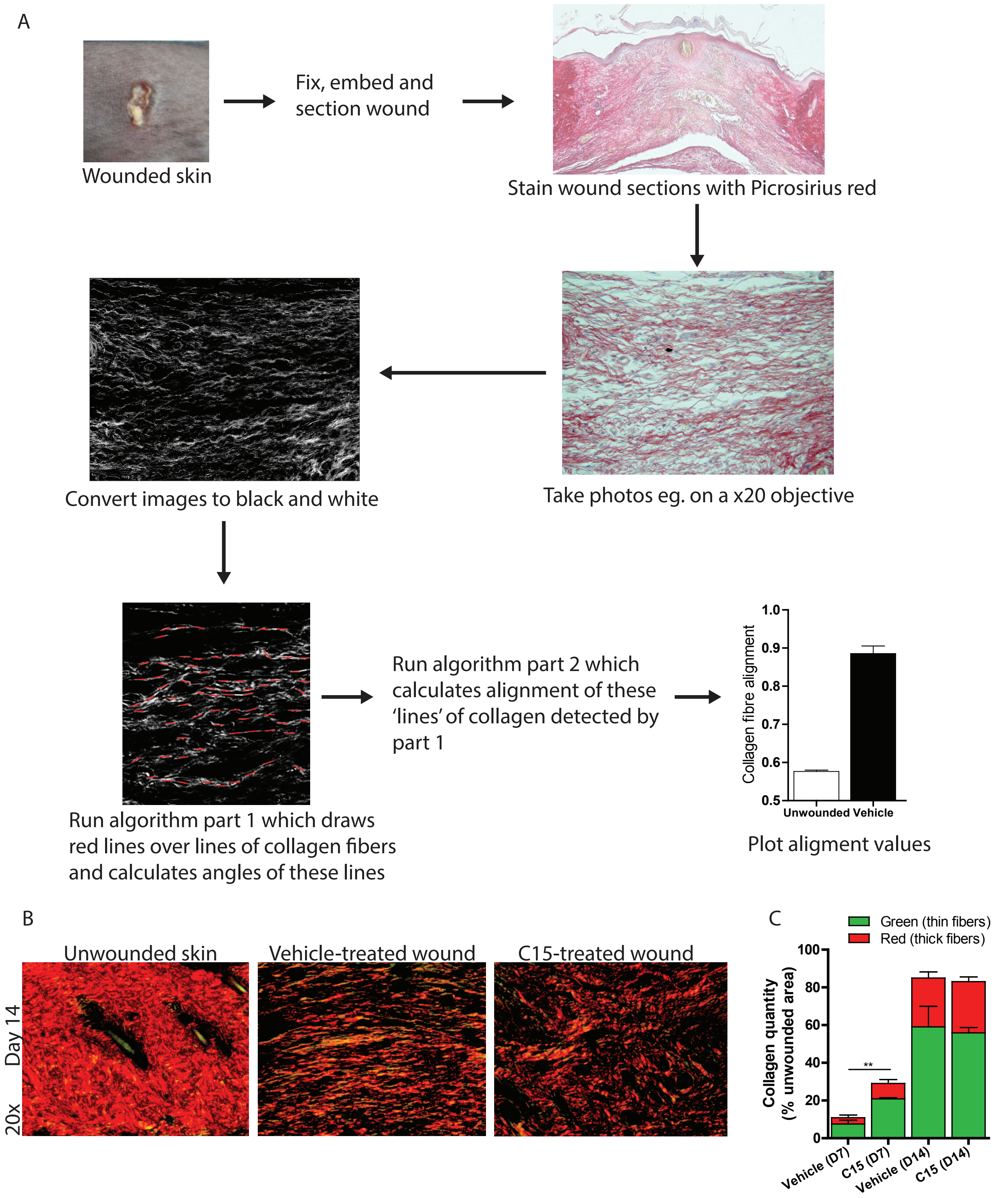

Figure S3 A Analysis of collagen alignment simplified as a flow diagram Two perpendicular lines have an alignment of 0, therefore in a random arrangement, total alingment = 0.5, and in perfectly aligned tissue, alignment = 1. **B Assessment of collagen quantity in granulation tissue during repair** Day 14 wound midsections from vehicle and C15-treated mice were stained with picrosirius red and viewed under polarised light. **C** Thick (red) and thin (green) fibers were quantified using ImageJ in day 7 and 14 wounds.
**, $p<0.01$ relative to vehicle treated wounds. See Fig. 4.

Supplemental Experimental Procedures

Preparation of Chemerin15 peptide

Scrambled C15 peptide (C15-S) was used in preliminary experiments to test whether PBS in pluronic gel would equally be an appropriate control. No change in wound contraction or neutrophil recruitment were observed with C15-S administration in this study. We used doses of 10 pg, 100 pg, 1 ng and 10 ng C15/wound in preliminary studies, finding that the 100 pg/wound dose achieved maximum acceleration in wound contraction and reduction in neutrophil recruitment, with no additional benefit observed with the higher doses of peptide. Peptide reconstitution was always performed under sterile conditions and reconstituted peptide is stable for approx. 6 months in -20C or -80C. Once thawed the peptide is stable on ice for several hours and is not photosensitive. When a peptide stock is thawed for use in wound repair assays it is diluted in sterile PBS and added to pluronic gel. The only step that becomes asterile is administration of the peptide to the wound. Sterile tips were used and the backs of the mice shaved and sprayed with ethanol, but wounding was not performed in aseptic conditions.

Preparation and immunohistological analysis of excisional cutaneous wounds

Wounds were harvested on days 1, 4, 7 and 14 post-wounding, fixed in 4% PFA (4 h at 4°C, Sigma) for embedding in paraffin. Day 1 and 4 wounds were cut and sections (10 μm) taken from the middle of the wound. Day 7 and 14 wounds were sectioned through the wound beyond the midpoint and wound centres identified by staining with Haematoxylin (Gills No.3) and Eosin (Sigma) and quantifying scab length (if present) and gap between severed edges of the panniculus carnosus using ImageJ software.

Proteinase K (5 μg/ml, 7 min; Dako) was used for antigen retrieval, except in the case of chemerin staining where heat-mediated antigen retrieval was performed (citrate buffer, Abcam; 95°C, 20 min). Avidin/Biotin kit, ABC kit, ABC-AP kit, DAB kit, Alkaline phosphatase Vector Blue kit, levamisole solution, biotinylated anti-rat, anti-goat and anti-rabbit secondary antibodies (all from Vector Laboratories; Peterborough, UK) were used according to the manufacturer’s instructions. Numbers of leukocytes per field of view (FOV) were calculated using thresholded 32-bit images and the “analyze particles” function in ImageJ software. Numbers of chemerin+ and ChemR23+ cells were quantified similarly.

Sections were deparaffinised, rehydrated and stained with Hematoxylin (10 s), Eosin (30 s), Toluidine Blue (mast cells; 20 min, Sigma Aldrich) or Picrosirius Red (collagen; 1 h), rat anti-

mouse F4/80 (monocyte-macrophages, BM8, 2.5 μg/ml; Ebiosciences), rat anti-mouse Ly6G (neutrophils; 1A8, 2.5 μg/ml, Biolegend), rat anti-mouse polyclonal CD31 (blood vessels, 5 μg/ml; BD Biosciences), rabbit anti-mouse TNFα (2.5 μg/ml, Abcam), goat anti-mouse Ym1 (0.8 μg/ml, R&D Systems), rabbit anti-mouse arginase 1 (4 μg/ml, Santa cruz), rabbit anti-mouse iNOS (1 μg/ml, Abcam), rabbit anti-mouse β-galactosidase (2 μg/ml, Invitrogen), rabbit anti-mouse α-SMA (smooth muscle actin, 0.06 μg/ml, clone E184, Abcam) and goat-anti mouse polyclonal chemerin antibody (2 μg/ml; R&D Systems) all overnight at 4°C.

Intravital imaging of cutaneous focal necrotic injury

Mice were anesthetized with a mixture of ketamine hydrochloride (200 mg/kg, Rogar/SBT) and xylazine hydrochloride (10 mg/kg, MTC Pharmaceuticals). After anaesthesia, the tail vein was cannulated for administration of additional anaesthetic and for injection of antibodies. Following a dorsal midline incision, the skin was dissected away from the underlying tissue yielding a skin flap that remained connected laterally with intact blood supply. The skin flap was extended over a viewing pedestal and immobilized with 4.0 silk sutures at three points around the edge of the flap. Loose connective tissue overlying the dermal microvasculature was carefully removed under a dissecting microscope.

After preparation for spinning disk intravital microscopy (SD-IVM) and immediately prior to imaging, a single 580±39 μm^2 focal injury was induced on the surface of the skin flap using the tip of a heated 30-gauge needle mounted on an electro-cautery device. C15 (100 pg) or vehicle were administered i.d immediately after wounding. For sham experiments, mice were prepared for SD-IVM and imaged identically to injured animals but no injury was induced. Following induction of necrotic injury the exposed dermal surface of the skin was covered with coverglass and continuously superfused with bicarbonate-buffered saline for the duration of the experiment. Mice were placed on a heating pad to maintain body temperature throughout experiments. Exposed tissues were visualized with an Olympus BX51 upright microscope equipped with a confocal light path (Wave-Fx; Quorum) based on a modified Yokogawa CSU-10 head (Yokogawa Electric Corporation) using a x4/0.16 UplanSApo objective or a x20/0.95W XLUMPPlanFI water immersion objective. Three laser excitation wavelengths (561-, 635- and 733 nm;Cobalt) were used in rapid succession and visualized with the appropriate long-pass filters (Semrock). Typical exposure times for excitation wavelengths were ~400 ms (561-nm), ~600 ms (635-nm) and ~900 ms (733-nm). A back-thinned EMCCD 512x512 pixel camera was used for fluorescence detection. Volocity software (Improvision) was used to drive the confocal microscope.

Neutrophils and platelets were visualized by intravenous administration of 2 μg eFLuor 660 conjugated Ly6G antibody (Ebioscience; 50-5931-82) or PE-CD49b antibody (BD Pharmingen; 558759) respectively. Blood vessels were visualized using 2 μg PerCP eFluor 710 conjugated CD31 (Ebioscience; 46-0311-82). Necrotic cells were labelled with a single application of 20 µL of 2 µM propidium iodide (Sigma Aldrich) solution to the wound.

For experiments investigating the quantity of neutrophils at sites of focal necrosis, images were acquired 2 h after injury using a 4x objective. The total number of neutrophils was then determined within specific regions of the microscopic field (within injury, 150 µm around injury, and 150-500 µm from injury border). To assess intravascular neutrophil-endothelial, platelet-endothelial and platelet-neutrophil interactions, 4-8 post-capillary venules (diameter 20-40 μm) were observed per skin prep per time point using a 20x objective and recorded for offline analysis. The extent of the inflammatory response was assessed by quantifying neutrophil rolling and adhesion, platelet rolling and adhesion and the number of neutrophils interacting with platelets in each treatment condition and time point.

Keratinocyte migration and proliferation assay

HaCaT cells (0.8 x10^6/ml) were plated into Ibidi inserts on nitric acid washed coverslips in a 24 well plate in 70 μl media (DMEM + 10% FBS, 1% Penicillin/Streptomycin). Cells were incubated for 4 h at 37°C, 5% CO_2, washed, the Ibidi insert removed and treatments (1 – 1000 pM C15) added in media. Cells were fixed 15 h later and gap closure assessed in ImageJ. Treatments were performed in sextuplicates.

Fibroblast-mediated collagen contraction assay

Murine dermal fibroblasts (a kind gift of Dr J Morris) were cultured in DMEM + 10% FBS (Gibco and Sigma) and harvested with 0.05% trypsin when 80% confluent. Cells (6 x10^4/well of a 4 well plate) were mixed with rat tail collagen I (Millipore), 10xRPMI (Gibco) and 238 mM $NaHCO_3$ (Sigma) to achieve final concentrations of 1.8 mg/ml, 1x and 24 mM respectively. Samples (600 μl) of the cell mixture were added to the wells of a 4-well tissue culture plate and spiked with 60 μl media control or C15 to achieve final concentrations of 10 pM, 100 pM and 1 nM. The collagen was allowed to polymerize at 37°C, after which the gels were gently detached from the plastic surface to allow contraction and 0.6 ml DMEM + 10% FBS containing 10 pM, 100 pM or 1 nM C15 added per well. The gels were incubated at 37°C, 5% CO_2 for 24 h and 48 h then photographs were taken.

Immunofluoresence of cultured cells

Primary dermal fibroblasts were cultured on acid washed glass coverslips coated with poly-l-lysine for 5 min. HaCaTs were cultured on glass coverslips coated with poly-l-lysine (5 min, Sigma) and coating matrix (30 min, Gibco). Cells were fixed with 4% PFA, washed with PBS, blocked with 10% goat serum for 30 min and incubated with rabbit anti-human ChemR23 (10 μg/ml, R&D Systems) or rat anti-mouse ChemR23 (20 μg/ml, Ebioscience) in 10% goat serum for 1 h at room temperature. Coverslips were washed with PBS and secondary antibody applied (goat anti-mouse Alexa 488 or donkey anti-rat Alexa 488) in 10% goat serum for 30 min. After washing coverslips with dH_2O, they were mounted in Prolong Gold Moviol containing DAPI and imaged with a confocal microscope. Controls were secondary only and isotype control with no appreciable staining detected.

Algorithm details for analysis of collagen alignment

Alignment is measured using the dot product. Each straight segment of the track defines a vector v_i, which may be compared to all the other segments of the track. The value of the dot product d_{ij} between vectors v_i and v_j is

$$d_{ij} = \frac{v_i \bullet v_j}{|v_i||v_j|}$$

If the vectors are parallel $d_{ij} = 1$, perpendicular gives $d_{ij} = 0$ and reversed gives $d_{ij} = -1$. The value of d_{ij} is then averaged over all segments, and the operation is in turn repeated for all N segments in the track to give

$$d = \frac{\sum_{i=1}^{i=N} \frac{\sum_{j=1}^{j=N} d_{ij}}{N}}{N}$$

Detailed instructions for use of the plugin to quantify collagen alignment.

(related to Figure 4 and Figure S3).

Please do not hesitate to contact jenna.cash@bristol.ac.uk and mark.bass@bristol.ac.uk for help if required.

Image processing

1) Stain wound sections with picrosirius red
2) Take eg. 20x photos of picrosirius red staining in wound granulation tissue (photos taken under polarised light should also work)
3) Save the Collalign plugin to the plugins folder in ImageJ (Disk C: -> Program Files -> Image J -> plugins)

4) Open the photo in ImageJ
5) Click Image -> Color -> Split channels. You will now have 3 images (red, green and blue)
6) There are 2 ways to generate the greyscale image for analysis. For most images, including sections only stained with picrosirius red use approach "a". Images with a counterstain may give better definition by calculating the contrast between picrosirius red and counterstain, use approach "b".

6a) i) Close red and blue images.
II) Select green image, click Edit -> Invert
iii) You will now have a black and white image of the collagen that looks something like the below.

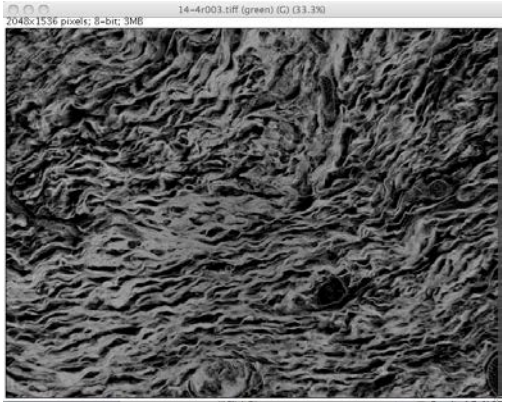

6b) i) Close green image.
ii) Click Process -> Image calculator -> subtract the blue image from the red image

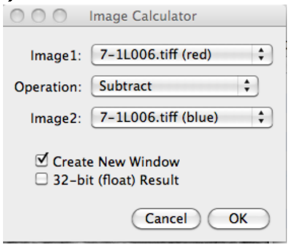

iii) You will now have a black and white image of the collagen that looks something like the below.

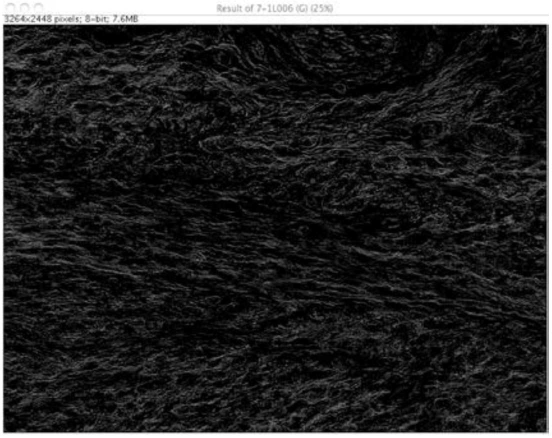

7) Click Plugins -> collagen alignment
8) The values for the presets suggested by the plugin will work for many images but can be altered if the plugin is not fitting lines well. The plugin works by scaling the

brightness of the image between minimum and maximum so should work with a range of image intensities. Adjustable variables include square size, overlapping area, mean grey value, minimal and maximum standard deviation of the grey levels. Each variable is defined in more detail at the end of the instructions.

9) The plugin will take 5-20 minutes to run, depending on the size of the image, the size of the boxes to be analysed, and whether you run overlapping boxes. You will see the analysis box moving over the image named ".tif". Each time the plugin successfully finds an intensity distribution can be added, the "orientation.tif" will come to the front and a red line will be added, and an angle value (in radians) will be added to the "log" file.

10) The "orientation.tif" image will automatically save to the location specified when starting the plugin. A zoom in of the orientation image will look like:

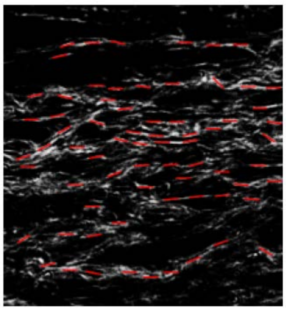

The red lines are centred in each square analysed and represent the angle of high intensity contrast across the whole square.

11) The log file will save to the same location, adding a new data set for each slice. The log file lists the angle (in radians) of each successfully fitted line. The results file is only required to run the plugin and not needed.

Alignment calculation

1) The alignment of fibres can be calculated by comparing the angle between individual lines, such that a pair of parallel lines will return an alignment value of 1 and a pair of perpendicular lines will return an alignment value of 0. This means that for perfectly aligned fibres, the total alignment value for parallel fibres will be 1 and for random fibres it will be 0.5.

2) Alignment can be calculated using the Bassalign.f90 fortran script.

3) Download and install the Silverlight compiler software: http://www.microsoft.com/silverlight/

4) From the computer programs list open:
Silverfrost -> Silverfrost FTN95 -> Plato IDE

5) In the Plato program, File -> Open -> Bassalign.f90

6) Build -> Compile

7) To create an input data file use notepad to a file in the format:

9		Where 9 is the number of data points.
1	0.0329	Where 1-9 are the sequentially numbered angle values to come out of the Collalign log file
2	0.0336	
3	-0.1201	
4	0.3294	
5	0.1237	
6	-0.0418	
7	0.1067	
8	0.3015	

9 0.5163

The numbers pasted across from the log file need to be followed by a couple of empty lines (hit return/enter twice).

8) Save the file as data.txt in the same place as the Bassalign.f90 file.
9) In the Plato program, Build -> Start run
10) The alignment value will be returned in a new window, along with the number of data values.

Adjustable variables are:

Square size: The plugin divides the image into squares and analyses each square in turn. Square size dictates size of the squares and can be set to 16, 32, 64, 128 or 256 pixels. Low values will find it easier to fit lines to fibres but are susceptible to noise in the image. Large values should in theory detect the strong fibres only, but often struggles to find a dominant line of intensity due to the high level of complexity in a single square. (Default = 64)

Overlapping area: The plugin divides the image into squares and analyses each square in turn. For overlap = 1, it will analyse divide up the image like a chessboard and analyse each square. For overlap = 2, the squares analysed will overlap so you will get more values from a single image, but there may be some redundancy as each pixel is being analysed twice.

***Mean grey value:** This instructs the plugin as to what value of mean grey value to expect in a square and stops the plugin fitting lines to noise values in very dim squares.

***Minimal standard deviation of the grey levels:** This ensures that the plugin is fitting lines to stained fibres, rather than noise in a square. Reducing the value will enable the plugin to find more squares, but generate more erroneous fits.

***Maximal standard deviation of the grey levels:** This stops the plugin from analysing artificial structures, such as air bubbles in the image. The brightness of genuine collagen fibres should fall within limited boundaries.

Parameters marked with an asterisk can be altered to improve sensitivity and fit more lines, or reduce erroneous fits.

Note: Up to 9 images can be converted into a single stack, and the plugin will analyse each slice in turn, saving the outputs as it goes.
